# Fe/Cu diatomic catalysts for electrochemical nitrate reduction to ammonia

**DOI:** 10.1038/s41467-023-39366-9

**Published:** 2023-06-19

**Authors:** Shuo Zhang, Jianghua Wu, Mengting Zheng, Xin Jin, Zihan Shen, Zhonghua Li, Yanjun Wang, Quan Wang, Xuebin Wang, Hui Wei, Jiangwei Zhang, Peng Wang, Shanqing Zhang, Liyan Yu, Lifeng Dong, Qingshan Zhu, Huigang Zhang, Jun Lu

**Affiliations:** 1 State Key Laboratory of Multiphase Complex Systems, Institute of Process Engineering, Chinese Academy of Sciences, Beijing, 100190 China; 2 National Laboratory of Solid State Microstructures, Collaborative Innovation Center of Advanced Microstructures, College of Engineering and Applied Sciences, Nanjing University, Nanjing, 210093 China; 3 College of Materials Science and Engineering, Qingdao University of Science and Technology, Qingdao, 266042 China; 4 Centre for Clean Environment and Energy and Griffith School of Environment, Griffith University, Gold Coast, QLD 4222 Australia; 5 Dalian National Laboratory for Clean Energy & State Key Laboratory of Catalysis, Dalian Institute of Chemical Physics, Chinese Academy of Sciences (CAS), Dalian, 116023 China; 6 Department of Physics, University of Warwick, Coventry, CV4 7AL UK; 7 School of Chemical Engineering, University of the Chinese Academy of Sciences, No. 19(A) Yuquan Road, Shijingshan District, Beijing, 100049 PR China; 8 College of Chemical and Biological Engineering, Zhejiang University, Hangzhou, Zhejiang Province 310027 China

**Keywords:** Electrocatalysis, Materials for energy and catalysis, Energy storage, Heterogeneous catalysis

## Abstract

Electrochemical conversion of nitrate to ammonia offers an efficient approach to reducing nitrate pollutants and a potential technology for low-temperature and low-pressure ammonia synthesis. However, the process is limited by multiple competing reactions and NO_3_^−^ adsorption on cathode surfaces. Here, we report a Fe/Cu diatomic catalyst on holey nitrogen-doped graphene which exhibits high catalytic activities and selectivity for ammonia production. The catalyst enables a maximum ammonia Faradaic efficiency of 92.51% (−0.3 V(RHE)) and a high NH_3_ yield rate of 1.08 mmol h^−1^ mg^−1^ (at − 0.5 V(RHE)). Computational and theoretical analysis reveals that a relatively strong interaction between NO_3_^−^ and Fe/Cu promotes the adsorption and discharge of NO_3_^−^ anions. Nitrogen-oxygen bonds are also shown to be weakened due to the existence of hetero-atomic dual sites which lowers the overall reaction barriers. The dual-site and hetero-atom strategy in this work provides a flexible design for further catalyst development and expands the electrocatalytic techniques for nitrate reduction and ammonia synthesis.

## Abstract (web summary)

Nitrate electroreduction to ammonia can decrease pollutants and produce high-value ammonia. Here, the authors design a Fe/Cu diatomic catalyst on nitrogen-doped graphene, which exhibits high catalytic activities of and selectivity for ammonia.

## Introduction

Ammonia is a common and important chemical in agriculture, plastic, pharmaceutical industries, etc^1,2^. Since the invention of the Haber–Bosch process, large-scale synthesis and application of ammonia dramatically increase the crop yield and sustain the growing human population^3,4^. However, the Harber-Bosch process consumes extravagant resources and energy (1–2% of the annual global energy supply) and produces 1% of CO_2_ emission (1.8 tons CO_2_ for 1 ton NH_3_)^5^, causing severe environmental impact. Therefore, clean and energy-efficient technologies for ammonia synthesis receive increasing attention. At the same time, human activities have ceaselessly released reactive nitrogen into the environment, leading to an imbalance in the global nitrogen cycle^6–8^. The increasing concentration of nitrate (NO_3_^–^) in surface water and underground aquifer pollutes the water resources and poses severe threats to human health^9–11^. As NO_3_^−^ is soluble and thermodynamically stable, removing NO_3_^–^ from water is considered a challenging and long-standing task^12,13^. The direct conversion of nitrate to ammonia could reduce environmental pollution and concurrently save energy for sustainable ammonia production.

Hydrogenation of nitrate to ammonia is intrinsically a spontaneous energy-releasing process (∆G < 0) because nitrates are usually strong oxidative agents^14^. To pursue carbon neutrality, a thermocatalytic nitrate reduction requires hydrogen gas (H_2_) to be produced in a green and clean way without CO_2_ emission (most industrial H_2_ is generated from fossil fuels by steam reforming of hydrocarbons or coal gasification). By contrast, the electroreduction of nitrate to ammonia (as follows) is a promising green strategy because electrons are clean reducing agents without producing secondary wastes^15,16^.

$${{{{{{{\mathrm{NO}}}}}}}}_{3}^{-}+6{{{{{{\mathrm{H}}}}}}}_{2}{{{{{\mathrm{O}}}}}}+8{e}^{-}\to {{{{{{{\mathrm{NH}}}}}}}}_{3}+9{{{{{{{\mathrm{OH}}}}}}}}^{-},\,{E}^{0}=0.69V\,{vs}\,{{{{{{\mathrm{RHE}}}}}}}({{{{{{\mathrm{pH}}}}}}}=14)$$

The electrochemical nitrate reduction reaction (NO_3_^−^RR) involves an eight-electron transfer, which has slow kinetics^17,18^. In addition, the competitive hydrogen evolution reaction (HER) and a variety of byproducts complicate the reaction routes and render the NO_3_^−^RR less selective and efficient^19,20^. To date, achieving efficient catalysts for the NO_3_^–^RR remains a major challenge mainly because the catalytic mechanism and the structure–activity relationship of catalysts are poorly understood^21^.

Single-atom catalysts (SACs) have recently emerged as a new frontier in catalysis science owing to their convenient atomic design, easy structure–activity correlation, and maximum atom utilization efficiency^22–24^. Tunable local coordination and well-defined active sites^25–27^ make SACs an ideal platform to study the structure–activity relationship of catalytic NO_3_^−^RR. By engineering the coordination environments of active single atoms, the energy barriers of different proton-electron transfer steps could be selectively shifted, offering opportunities of increasing the selectivity of the NO_3_^–^RR. Sargent et al. reported that the appropriately optimized d-band center position could enhance the NO_3_^−^RR performance of Cu_50_Ni_50_ alloy as compared to pure Cu^28^. Guo et al. loaded a single transition metal atom on graphitic carbon nitrides and demonstrated efficient nitrate degradation and ammonia synthesis^14^. Fe SACs were reported to have high activity and selectivity toward the production of NH_3_ via the NO_3_^–^RR^29^. Yu et al. fabricated isolated Fe sites in FeN_4_ coordination and displayed twelve times higher turnover frequency than Fe nanoparticles^30^. These important studies imply that SACs including Fe/Cu may offer higher catalytic behavior. However, for multi-electron transfer reactions, one specific site makes SACs difficult to break the linear scaling relations of adsorption strength between catalysts and multiple similar intermediates^31,32^. Optimizing the interaction of a key intermediate in the rate-determining step (r.d.s) may turn another step to the r.d.s^33^. Dual-site catalysts could extend the catalysts with substantially different coordination environment^34,35^. However, the precise design and fabrication of dual-site catalysts remain a challenge and the catalytic mechanism of heteroatomic sites is elusive, especially for NO_3_^–^RR.

**Fig. 1:**
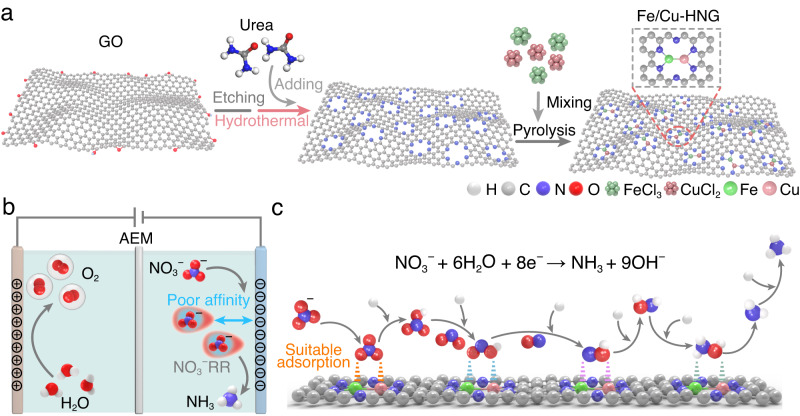
Schematic illustration of the synthesis of Fe/Cu-HNG and electrochemical nitrate reduction. **a** Schematic illustration of catalyst construction. **b** Electrochemical nitrate reduction. **c** Catalytic conversion steps from NO_3_^−^ to NH_3_.

In this work, we propose a dual atoms catalyst for high-efficiency NO_3_^–^RR. Active metal atoms (Fe/Cu) are anchored to the holey edge sites of nitrogen-doped graphene (HNG), forming a “Y-type” ML_3_ coordination with two nitrogen atoms and one metal atom. Fe and Cu atoms bind together to form a dimer structure inside the holes (Fig. 1a). The resultant catalyst, Fe/Cu-HNG, demonstrates high activity (~38.5 mA cm^−2^ at −0.3 V vs reversible hydrogen electrode (RHE)) and selectivity (maximum Faradaic efficiency (FE) of 92.51%) for the reduction of NO_3_^–^ towards NH_3_ under alkaline conditions. The maximum NH_3_ yield rate is as high as 1.08 mmol h^−1^ mg^−1^ at −0.5 V vs RHE. By combining operando differential electrochemical mass spectrometry (DEMS) and density functional theory (DFT) calculations, we reveal the reaction pathways and conversion mechanisms from NO_3_^−^ to NH_3_. An in-depth analysis of electronic structure indicates that the strong coupling between NO_3_ and d-orbitals of dual metal atoms lowers the energy barrier of the first anionic adsorption step, which is the r.d.s at high current densities. The dual atoms heterostructure could further weaken N-O bonds, enabling low energy barriers for NH_3_ production. The synergistic effects of dual atoms offer an alternative approach to designing the NO_3_^–^RR catalysts.

## Results

### Structural characterization of Fe/Cu-HNG

To anchor dual atoms and form metal–metal dimers (as shown in Fig. 1a), we first engineered holes in graphene to create a great number of edge sites, which were further nitrified to bind Fe/Cu atoms. Holey graphene was fabricated by sonicating graphene oxide (GO) in nitric acid (68%). The strong oxidation capability of nitric acid would cut the C-C bonds of GO layers to yield epoxy chains (carboxyl and/or hydroxyl) and other defects^36–38^. During the following hydrothermal and annealing treatments, the above-mentioned functional groups and defects were substituted by nitrogen, forming HNG. Supplementary Fig. 1 shows the scanning electron microscope (SEM) images of HNG and reduced GO (rGO). An rGO layer exhibits a large and flat area whereas HNG becomes porous. The transmission electron microscope (TEM) images in Supplementary Fig. 2 further reveal the successful synthesis of micropores on HNG. When Fe/Cu precursors were added during the hydrothermal and annealing steps, Fe/Cu dual atoms could be loaded onto the N-edge of micropores. The Fe/Cu loadings in Fe/Cu-HNG are determined to be 3.3 and 2.8 wt%, respectively, by using an inductively coupled plasma method (Supplementary Table 1).

**Fig. 2:**
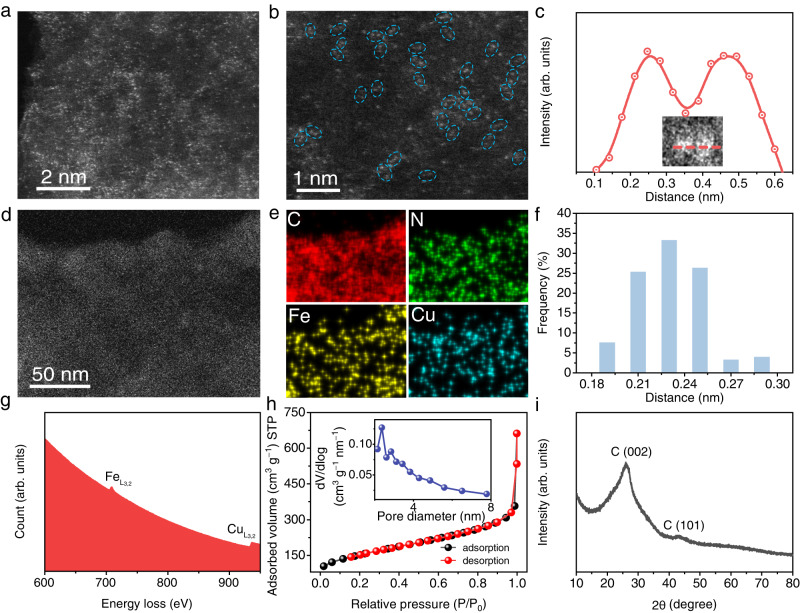
Materials characterization of Fe/Cu dual atoms catalyst. **a** HAADF-STEM image of Fe/Cu-HNG. **b** Zoomed-in HAADF-STEM image indicates the formation of dual atoms sites where two distinct adjacent bright dots were marked with blue dashed circles. **c** Corresponding intensity profiles of dual atoms pair. **d** STEM and **e** EDX elemental mapping images of C, N, Fe, and Cu in Fe/Cu-HNG. **f** Statistical distribution of Fe/Cu distance of the observed diatomic pairs. **g** EELS spectrum of Fe/Cu atomic sites. **h** N_2_ adsorption–desorption isotherms of Fe/Cu-HNG (inset: corresponding pore-size distribution). **i** XRD pattern of Fe/Cu-HNG.

Figure 2a, b show the aberration-corrected high-angle annular dark-filed scanning TEM (HAADF-STEM) images of Fe/Cu-HNG. A great number of atom-sized bright dots are distributed on HNG. By zooming into the image, diatomic pairs (like dimers) could be mostly observed. Energy-dispersive X-ray spectroscopic (EDX) line scan through two bright dots indicates a distance of ~2.3 Å (Fig. 2c), which is in agreement with the bond length of Fe-Cu in the simulated model (Fig. 1a). The EDX elemental mapping images in Fig. 2d, e clearly indicate the uniform distribution of C, N, Fe, and Cu. Figure 2f presents a statistical distribution of the metal–metal pair length. The average bond length is 2.3 ± 0.2 Å. The electron energy loss spectrum (EELS) in Fig. 2g reveals the coexistence of Fe and Cu elements in Fe/Cu-HNG. More complementary characterizations and statistical analyses were shown in Supplementary Figs. 3, 4 to identify diatomic sites.

The N_2_ adsorption–desorption isotherms of Fe/Cu-HNG (Fig. 2h) confirm the presence of highly mesoporous structures. The corresponding pore-size distribution has a major peak of 2–3 nm. The Brunauer–Emmett–Teller (BET) method is used to estimate the surface area to be 858 m^2^ g^−1^, which is five times higher than rGO (132 m^2^ g^−1^, Supplementary Fig. 5). A high surface area and porous structure could facilitate mass transport and improve the apparent activity of catalysts^39–41^. The X-ray diffraction (XRD) pattern (Fig. 2i) shows only two broad peaks, which are ascribed to stacking graphene layers (Supplementary Fig. 6)^42,43^. No other impurity peaks are observed. In conjunction with a wide-range SEM (Supplementary Fig. 7) and TEM (Supplementary Fig. 8) analysis, we may conclude that Fe/Cu diatomic structures were successfully constructed on HNG and no aggregation or nanoparticles of metals were observed. For comparison, homogenous diatomic materials (Fe/Fe-HNG and Cu/Cu-HNG) were also synthesized by adding Fe or Cu precursors, respectively. Detailed morphologic and structural characterizations of Fe/Fe-HNG and Cu/Cu-HNG were shown in Supplementary Figs. 9,10, respectively.

**Fig. 3:**
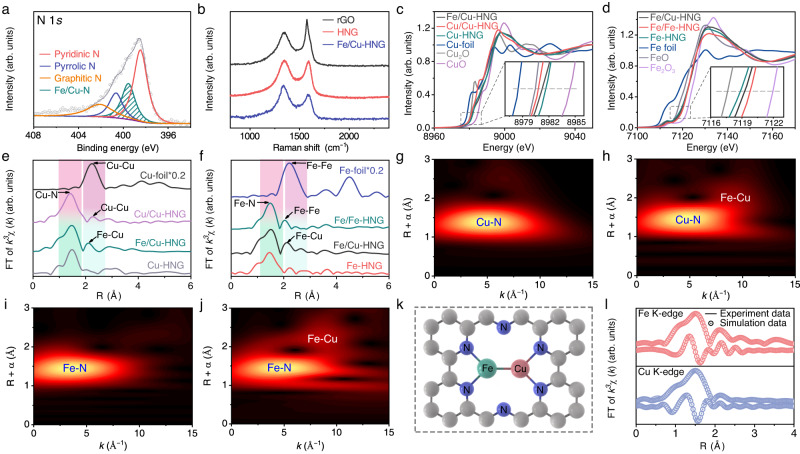
Atomic structural and chemical states analyses of Fe/Cu dual atom catalyst. **a** High-resolution N 1*s* XPS spectrum of Fe/Cu-HNG. **b** Raman spectra of rGO, HNG, and Fe/Cu-HNG. **c** Cu K-edge XANES spectra of Fe/Cu-HNG, Cu/Cu-HNG, Cu-HNG, Cu foil, CuO, and Cu_2_O. **d** Fe K-edge XANES spectra of Fe/Cu-HNG, Fe/Fe-HNG, Fe-HNG, Fe foil, FeO, and Fe_2_O_3_. **e** *k*^3^-weighted FT of χ(k)-function from the Cu K-edge EXAFS. **f** *k*^3^-weighted FT of χ(k)-function from the Cu K-edge EXAFS. WT images of the Cu K-edge from **g** Cu-HNG **h** Fe/Cu-HNG, and the Fe K-edge from **i** Fe-HNG, and **j** Fe/Cu-HNG. **k** Proposed schematic model of Fe/Cu-HNG: Fe (aqua), Cu (orange), N (blue), and C (brown). **l** Fitting results of the EXAFS spectra of Fe/Cu-HNG at k-space and R space of Fe K-edge.

Figure 3a presents the X-ray photoelectron spectroscopic (XPS) analyses of the N 1*s* signal. The broad peak of the N 1*s* signal could be de-convoluted to pyridinic N (~398.3 eV), pyrrolic N (~400.6 eV), graphitic N (~401.4 eV), and Fe–N/Cu–N (~399.0 eV)^44,45^. The Fe 2*p*_*3/2*_ signals exhibit two peaks that could be assigned to the Fe^2+^ (709.9 eV) and Fe^3+^ (711.5 eV)^46,47^. By decreasing the number of metal precursors, we synthesized single-atom-loaded HNG (labeled as Fe-HNG or Cu-HNG,). As compared to single-atom Fe-HNG, the Fe 2*p* peaks of Fe/Cu-HNG shift toward high binding energy (Supplementary Fig. 11b). The Cu 2*p*_*3/2*_ spectrum in Supplementary Fig. 11c shows two peaks at 932.5 and 933.7 eV, which are assigned to Cu^+^ and Cu^2+^, respectively^48,49^. By contrast, the Cu 2*p* peaks of Fe/Cu-HNG have lower binding energy than those in single-atom Cu-HNG. The XPS peak shift of Fe/Cu dual atoms may imply electron transfer from Fe to Cu. The Raman spectra in Fig. 3b show two typical D and G bands of graphene at 1362  and 1576 cm^−1^, respectively. The intensity ratio of D to G is higher for Fe/Cu-HNG than that for rGO and HNG, indicating that the defective carbon nanosheets are formed owing to Fe/Cu dopants^50,51^.

Figure 3c–f show the X-ray absorption near-edge spectra (XANES) and extended X-ray absorption fine structure (EXAFS). The Cu K-edge XANES of Fe/Cu-HNG (Fig. 3c) confirms that Cu has an oxidation state between Cu_2_O (1+) and CuO (2+)^52^. Similarly, the Fe K-edge XANES of Fe/Cu-HNG (Fig. 3d) resides between Fe (0) foil and Fe_2_O_3_ (3+), indicative of the oxidized Fe in Fe/Cu-HNG^43,53^. As compared to Fe-HNG, a minor shift of Fe K-edge in Fe/Cu-HNG toward high energy implies that Fe/Cu dimers in HNG slightly increase the oxidation state of Fe owing to Cu ligands. Fe atoms in Fe/Cu-HNG transfer electrons to Cu and slightly reduce Cu as compared to Cu-HNG. Such a trend obtained by the absorption spectra is in agreement with the XPS analyses. The *k*^3^-weighted Fourier transform (FT) from Cu K-edge EXAFS spectra (Fig. 3e) show that the major peaks of Fe/Cu-HNG, Cu-HNG, and Cu/Cu-HNG are located at ~1.45 Å, which corresponds to the first shell scattering of the Cu–N coordination^54^. Notably, the second peaks at 2.15 and 2.27 Å for Fe/Cu-HNG and Cu/Cu-HNG, respectively, are comparable to the first shell distance of Cu foil (2.24 Å), suggesting the presence of metal–metal diatomic configuration. Similarly, the major peaks at ~1.48 Å for Fe/Cu-HNG, Fe-HNG, and Fe/Fe-HNG in Fig. 3f are ascribed to Fe–N coordination^55,56^. The second peaks at 2.15 and 2.03 Å for Fe/Cu-HNG and Fe/Fe-HNG, respectively, further confirm the presence of metal–metal diatomic configuration. The *k*^3^-weighted FT spectra indicate that the metal–metal distance in Fe/Cu-HNG is shorter than Cu–Cu coordination in Cu/Cu-HNG and longer than Fe–Fe coordination in Fe/Fe-HNG, verifying the existence of heterogeneous Fe-Cu sites in Fe/Cu-HNG. Figure 3g–j present the wavelet transform (WT) of the EXAFS spectra. Both Cu-HNG and Fe/Cu-HNG exhibit the intensity maxima at ~4.8 Å^−1^ due to the Cu–N path in the Cu K-edge spectra, differing from the Cu–O path (~4.2 Å^−1^). In addition, Fe-HNG and Fe/Cu-HNG display the intensity maxima at 4.6 Å^−1^ due to the Fe–N path in the Fe K-edge spectra. For Fe/Cu-HNG, the WT of either Fe or Cu K-edge signals display a maximum at 2.15 Å, which is between the metal–metal bond length of Cu and Fe foils and different with Fe/Fe-HNG and Cu/Cu-HNG (see Supplementary Figs. 9–13), suggesting the formation of Fe–Cu bond (Fig. 3h, j). Therefore, the WT and FT-EXAFS analyses confirm the existence of metal–N coordination and metal–metal bonds in Fe/Cu-HNG^57–59^.

To further verify the coordination structure of Fe/Cu-HNG, we used the model in Fig. 3k to fit the FT-EXAFS curves in Fig. 3l (see other fittings in Supplementary Figs. 9–14). The fitted main peak around 1.97 Å and 2.05 Å originate from the first Fe–N and Cu–N coordination shell. The coordination numbers of Fe–N and Cu–N are around 2.16 and 2.23 (Supplementary Table 2), respectively. The second peak in Fig. 3l is fitted to be ~2.25 Å, which corresponds to the Fe–Cu path. The good agreement between the experimental and fitting results confirms the proposed structure that Fe/Cu dual atoms are anchored on MN_2_ sites and the neighboring Fe/Cu atoms bond together to form a metal–metal dimer structure^60,61^.

### Electrocatalytic performance for the NO_3_^−^RR

**Fig. 4:**
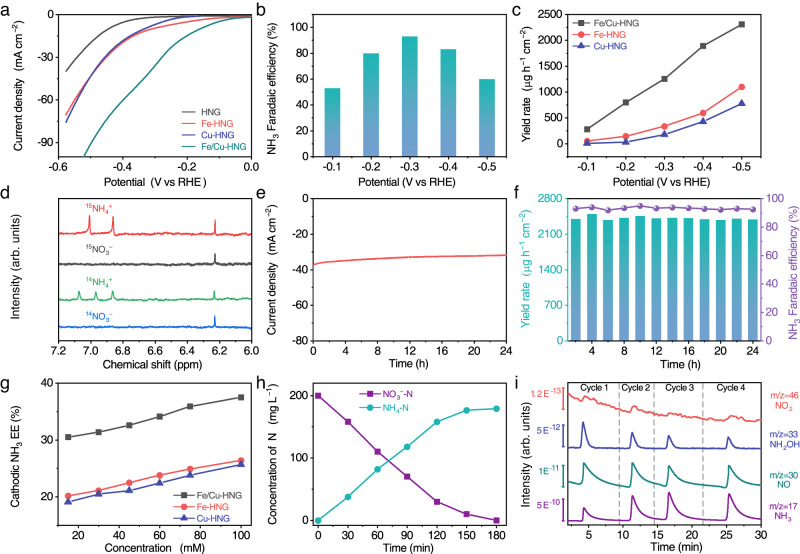
Electrochemical properties and mechanism of NO_3_^−^RR ammonia synthesis. **a** LSV curves of Fe/Cu-HNG, Fe-HNG, Cu-HNG, and HNG in an electrolyte of 1 M KOH and 0.1 M KNO_3_. **b** NH_3_ FEs of Fe/Cu-HNG at varied potentials. **c** NH_3_ yield rates of Fe/Cu-HNG, Fe-HNG, and Cu-HNG. **d** ^1^H NMR spectra of the electrolytes after electrocatalysis at −0.3 V (vs RHE) using 0.1 M ^15^NH_4_^+^ or 0.01 M ^14^NH_4_^+^ in 1 M KOH as nitrogen source (^1^H NMR of the fresh electrolytes marked as ^15^NO_3_^−^ and ^14^NO_3_^−^ were provided as the controls). **e** Chronoamperometric curve of Fe/Cu-HNG at −0.3 V vs RHE for 24 h. **f** Cycling tests of Fe/Cu-HNG for the NO_3_^−^RR. **g** Comparison of the cathodic NH_3_ EEs obtained using the Fe/Cu-HNG, Fe-HNG, and Cu-HNG catalysts. **h** Time-dependent concentration changes of NO_3_^−^ and ammonia during the NO_3_^−^RR using Fe/Cu-HNG. **i** DEMS analyses of nitrogen species during the NO_3_^−^RR (each cycle is one LSV scan from 0.1 to −0.6 V vs RHE).

Figure 4a presents the linear sweep voltammetry (LSV) curves of electrochemical nitrate reduction in an electrolyte of 1 M KOH and 0.1 M KNO_3_. HNG delivers ultralow current density until the negative polarization reaches −0.4 V vs RHE. SACs (Fe-HNG and Cu-HNG) slightly push the on-set potential to positive voltages and modestly increase the current density. Furthermore, by increasing metal loading, we synthesized homogenous diatomic catalysts (Fe/Fe-HNG and Cu/Cu-HNG). Supplementary Fig. 15 shows that dual sites further improve the catalytic activity. Notably, hetero-atomic dual-site catalyst (Fe/Cu-HNG) dramatically boosts the current density as compared to single-atom, homogenous diatomic catalysts, and a mechanic mixture of Fe/Fe-HNG and Cu/Cu-HNG. In particular, the LSV curve of Fe/Cu-HNG exhibits a downward hump around −0.3 to −0.5 V vs RHE, which may result from the mass-transport-limited reduction of nitrates. To separate the contribution of NO_3_^−^RR from HER, we first measured the LSV in 1 M KOH without 0.1 M KNO_3_ (Supplementary Fig. 16). Supplementary Fig. 17a shows that Fe/Cu-HNG does not catalyze the HER well and delivers a nearly zero current density (at voltages higher than −0.35 V) in a pure KOH solution, implying a low contribution of the HER to the total current density in the nitrate solution. Given that the HER and NO_3_^−^RR are competitive reactions, we tentatively simulated the total reactions by simultaneously considering the HER with the Butler-Volmer equation and the NO_3_^−^RR with a mass-transport-limited LSV relation. Supplementary Fig. 17b shows that the NO_3_^−^RR accounts for 85.7% of total transferred electrons in the LSV measurement.

To confirm the LSV analytic results, we quantified the Faraday efficiency (FE) and yield rate of major products at varied voltages (Eqs-1, 2 in Supplementary Methods) using standard curves of NH_3_, NO_3_^−^, and NO_2_^−^ in Supplementary Figs. 18–20. In addition, a typical electrolysis curve and UV-vis testing curves confirm the increase in NH_3_ (Supplementary Fig. 21). Figure 4b demonstrates that the FE of NH_3_ initially increases with voltages and then decreases at high voltages. Specifically, Fe/Cu-HNG enables the higher FE maximum of NH_3_ (92.51%) at a more positive voltage (−0.3 V) than SACs Fe-HNG and Cu-HNG (Supplementary Figs. 22,23), suggesting that diatomic catalysts have higher catalytic activity than SACs. The yield rates of NH_3_ displayed in Fig. 4c further exemplify the much-enhanced activity and selectivity of Fe/Cu-HNG as compared to Fe-HNG and Cu-HNG. Supplementary Table 3 summarizes the performance of previously-reported catalysts. Fe/Cu-HNG delivers high yield rates (1.08 mmol h^−1^ mg^−1^ at −0.5 V vs RHE) and the ultralow energy consumption (8.76 Wh g_NH3_^−1^ mg^−1^), demonstrating the high catalytic activity of diatomic sites. To determine the N source of the detected ammonia and assess the yield rate of NH_3_ independently, a ^1^H nuclear magnetic resonance (NMR) test was employed to identify the NH_3_ generation of Fe/Cu-HNG in 1 M KOH with 0.1 M ^15^NO_3_^−^ or ^14^NO_3_^−^ (Fig. 4d and Supplementary Fig. 24). The typical ^1^H NMR spectra show two peaks due to ^15^NH_4_^+^ after electrolyzing ^15^NO_3_^−^ and three peaks related to ^14^NH_4_^+^ after electrolyzing ^14^NO_3_^−^, confirming that the product NH_3_ actually originates from the NO_3_^−^RR rather than contaminations^62^. The ^15^NH_3_ and ^14^NH_3_ yields were quantified by the averaged NMR peak areas. The calibration curves of ^1^H NMR spectra are in good agreement with UV-vis spectrophotometry measurements by colorimetric methods, demonstrating the reliability of the ammonia production efficiency test (Supplementary Fig. 24c). The FEs of byproducts about NO_2_^−^ and H_2_ were shown in Supplementary Fig. 25.

Figure 4e presents the chronoamperometric curve. Only a slight decrease in the current density is observed at a constant voltage. Continuous electrolysis for 24 h (Fig. 4f) shows that the FEs and yield rates of NH_3_ could be maintained at 90% and ~2400 μg h^−1^ cm^−1^, respectively, indicative of the electrochemical stability of Fe/Cu-HNG catalysts. The HAADF-STEM analyses indicate that the diatomic sites in Fe/Cu-HNG remain after 24 h (Supplementary Fig. 26). The distribution of diatomic sites is similar to that prior to the test. Furthermore, the *k*^3^-weighted FT of χ(k)-function in Supplementary Fig. 27 indicates the metal–N coordination and metal–metal remain similar as well. Supplementary Fig. 28 presents the LSV curves after 24 h cycles. The voltage curve nearly overlaps with that prior to the test, suggesting the stability of the Fe/Cu-HNG catalyst. Figure 4g displays the energy efficiency (EE) of NH_3_ production at varied concentrations (Eq-3 in Supplementary Methods). Owing to the lower overpotentials, Fe/Cu-HNG demonstrates much higher EEs than Fe-HNG and Cu-HNG. Supplementary Fig. 29 provides the influence of nitrate concentration on the NH_3_ yield rates and FEs. The maximal FEs of NO_3_^–^ to NH_3_ in the tested concentration range were 83–93% at −0.3 V (vs RHE). Fe/Cu-HNG basically exhibits appreciable ammonia yield rates and high selectivity under varied nitrate concentrations. Figure 4h presents the concentration changes of NO_3_^−^-N and NH_3_-N in H-cell batch electrolysis (50 mL). Fe/Cu-HNG (1 × 1 cm^2^) could almost completely reduce 200 mg L^−1^ NO_3_^−^-N and generate 189.2 mg L^−1^ NH_3_-N in 180 min. The sum of NO_3_^−^-N and NH_3_-N is less than the initial NO_3_^−^. The imbalance of N implies the existence of byproducts beyond NH_3_.

To decipher the intermediate byproducts and reaction pathway, we conducted a DEMS analysis for multiple cycles^63,64^ (see the schematic setup in Supplementary Fig. 30). During each cycle, the applied voltage was scanned from 0.1 to −0.6 V (vs RHE). Figure 4i presents the mass-to-charge (m/z) ratio signals of 46, 30, 33, and 17, which correspond to NO_2_, NO, NH_2_OH, and NH_3_, respectively. In addition to the major product (NH_3_), NO has two orders of magnitude higher fraction than NH_2_OH and NO_2_.

#### Theoretical analysis of NO_3_^−^RR mechanism

**Fig. 5:**
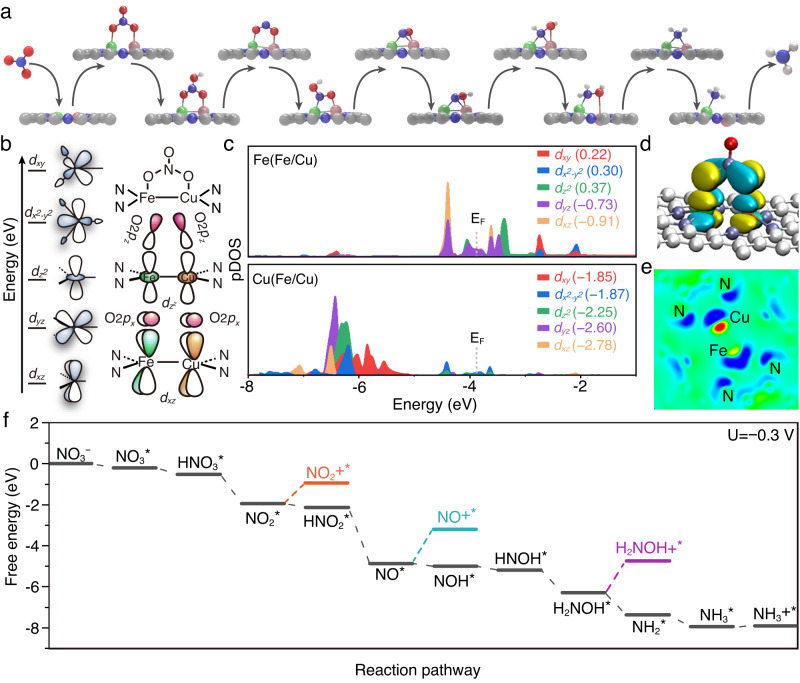
Theoretical analyses of the catalytic mechanism. **a** Reaction pathway of the NO_3_^−^RR and adsorption models of intermediates. **b** MO theory analysis of the splitting of metal 3*d* orbitals in a “Y-type” triple coordination and the interaction diagram between Fe/Cu 3*d* orbitals and O*p* orbitals of NO_3_. **c** PDOS of Fe/Cu 3*d* orbitals. **d** Interacting Wannier orbitals of NO_3_ on Fe/Cu-HNG. **e** Electron density difference diagram in the sliced plane through the Fe/Cu dimer. **f** Free energy diagram of each intermediate state on the metal atom sites in Fe/Cu at U = −0.3 V vs RHE.

There exist four possible pathways^65,66^ from NO_3_^−^ to NH_3_ as shown in Supplementary Fig. 31. The DEMS measurements reveal the appearance of NO_2_, NO, and NH_2_OH, implying the most possible reaction pathway as illustrated in Fig. 5a. NO_3_^−^ is first adsorbed and discharged to form *NO_3_, which plays an important role because the poor affinity of NO_3_^−^ make the discharge difficult (Fig. 1b). Once absorbed, *NO_3_ is then hydrogenated to form *NO_3_H, which is further attacked by protons to release H_2_O and yield *NO_2_. The hydrogenation/dehydration cycle reduces *NO_2_ following the sequence: *NO_2_H → *NO → *NOH → *NHOH → *NH_2_OH → *NH_2_ → *NH_3_. The last step is the desorption of NH_3_ off catalysts.

To understand the catalytic mechanism of the NO_3_^−^RR, we first analyzed the structure and bonding of Fe/Cu-HNG and the influence of diatomic sites on the reaction routes. The geometric model of Fe/Cu-HNG is constructed from the EXAFS fitting result (Fig. 3k). Based on the molecular orbital (MO) theory understanding of “Y-type” ML_3_ coordination (see discussion below Supplementary Fig. 32), we plotted the energy level splitting of 3*d* orbitals and their interactions with NO_3_ in Fig. 5b. Figure 5c shows the calculated partial density of states (PDOS) of *d*-orbitals of Fe/Cu-HNG. By integrating these 3*d* orbitals, we obtained the relative energy level of each d-orbital. Their relative positions are in agreement with the MO theory analysis in Fig. 5b.

Furthermore, NO_3_ on Fe/Cu-HNG was modeled and analyzed. After geometric optimization, two oxygen atoms of NO_3_ are attracted to Fe/Cu dual sites. Figure 5d presents the interacting Wannier orbitals of NO_3_ on Fe/Cu-HNG, where the 3*d*_*xz*_ orbitals of Fe/Cu form bonds with 2*p*_*x*_ orbitals of two oxygen of NO_3_, which basically agree with the MO theory analysis. The binding energy of NO_3_ on Fe/Cu-HNG is −1.19 eV, which is stronger than that of single-atom sites (−0.89 eV for Fe-HNG and −0.56 eV for Cu-HNG, Supplementary Fig. 33). Strong adsorption of NO_3_ could lower the energy barrier of the first discharge step (${ \ast+{{{{{{\mathrm{NO}}}}}}}}_{3}^{-}{\to {{{{{{\mathrm{NO}}}}}}}}_{3}^{*}+{e}^{-}$). NO_3_^−^RR differs from other electroreduction reactions (like the HER) because the planar symmetrical (D_3h_) resonant structure of NO_3_^−^ and a strong hydrogen bond with water weaken the interaction between NO_3_^−^ and electrodes and thus limits electron transfer^67,68^. Once absorbed, negative polarization will accelerate the following hydrogenation and/or dehydration steps. Diatomic sites facilitate the first discharge step owing to the strong adsorption to NO_3_ and however, may increase the energy barriers of the following steps because other intermediates may also strongly bind to the diatomic sites.

An efficient catalyst has to make a balance between the need for strongly adsorbing NO_3_ and modestly adsorbing other intermediates. Furthermore, we analyzed the adsorption configuration and energy change of intermediates on diatomic sites. Supplementary Fig. 34 presents the optimized geometric models of *NO_3_ on these diatomic sites. The binding energy of *NO_3_ on Fe/Fe is −1.89 eV, which is stronger than that on Cu/Cu (−0.21 eV) because the Fe $3{d}_{{z}^{2}}$ and 3*d*_*xz*_ states have a higher level than those of Cu. In addition, it is noted that the Fe-O bond length of *NO_3_ on Fe/Fe is 1.823 Å and the Cu-O bond length of *NO_3_ on Cu/Cu is 1.945 Å, implying that Fe interacts more strongly with intermediates than Cu. Supplementary Fig. 35 shows that most intermediates (except *NO_2_ and *NH_2_) on Fe/Cu-HNG have medium binding energies as compared to them on Fe/Fe-HNG or Cu/Cu-HNG. It is understandable from the viewpoint of the d-band center that higher 3*d* orbitals of Fe than Cu may lead to stronger adsorption of most intermediates. The exceptions may mainly result from hetero-atomic dimer configuration, which is also demonstrated by the electron density difference diagram in Fig. 5e. Fe transfers electrons to Cu, forming a polarized metal–metal dimer. Such a Fe/Cu hetero-atomic dimer tunes the adsorption energy slightly off the general trend.

Supplementary Fig. 36 presents the energy diagram at the equilibrium potential (U = 0.69 V). The second step (*NO_3_ → *NO_3_H) has the highest energy barrier at equilibrium. When the voltage is polarized to −0.3 V vs RHE (Fig. 5f), all the steps are downhill and the NO_3_^−^RR occurs spontaneously. Therefore, the diatomic sites of Fe/Cu-HNG appropriately reconcile the conflicting requirements on reducing energy barriers of both the initial discharge and following hydrogenation/dehydration steps, thereby dramatically enhancing the catalytic activity of NO_3_^−^RR from NO_3_^−^ to NH_3_.

**Fig. 6:**
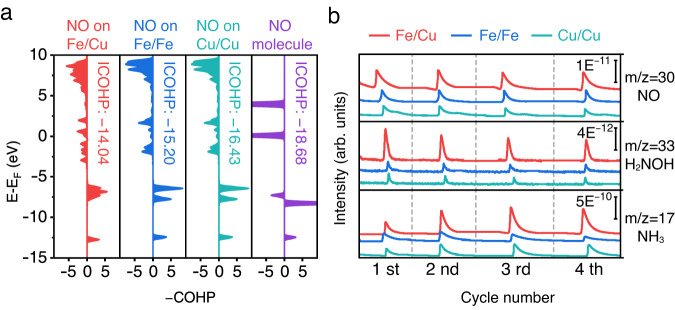
Theoretical and experimental analyses of N-O bond activation. **a** Crystal orbital Hamilton population (−COHP) and its integrated value (ICOHP) of NO* adsorption on different metal sites. **b** DEMS analyses of hydrogenation intermediates after the NO* adsorption step during the NO_3_^−^RR.

To further elucidate the subsequent deoxygenation/hydrogenation, we calculated the crystal orbital Hamilton population (COHP) of NO molecules adsorbed on Fe/Cu, Fe/Fe, and Cu/Cu diatomic sites. The integrated COHP (ICOHP) in Fig. 6a can be used as a quantitative indicator of the N-O activation. Compared to a free NO molecule, NO molecules on Fe/Fe and Cu/Cu sites were activated to varying degrees. A relatively more positive ICOHP (−14.04 eV) of NO molecules on Fe/Cu sites indicates a substantially weakened N-O bond because of the hetero-atomic structure. The activated N-O bond will facilitate the subsequent hydrogenation^69^. Operando DEMS measurements were conducted to verify the calculation results. Figure 6b shows that the NO yield ratio of Fe/Cu to Cu/Cu (or Fe/Fe) is around 2. Owing to the significant activation of Fe/Cu sites, the NH_2_OH/NH_4_ yield of Fe/Cu-HNG is dramatically increased to 8–10 times higher than those of Cu/Cu or Fe/Fe catalysts. It should also be noted that the intensity scale of the NH_3_ signal is two orders of magnitude higher than intermediates. These experimental and theoretical results confirm that Fe/Cu-HNG could lower the energy barrier of NO_3_^−^RR and activate NO molecules, leading to a high NH_3_ yield and selectivity.

## Discussion

Electrochemical reduction of nitrate to ammonia could reduce nitrate pollution and concurrently realize low-temperature and low-pressure ammonia synthesis. The slow kinetics of the nitrate-to-ammonia reaction requires efficient catalysts. We synthesized a Fe/Cu diatomic catalyst on holey nitrogen-doped graphene for nitrate reduction. Fe/Cu is coordinated with two nitrogen atoms and one metal, which is similar to a “Y-type” ML_3_ structure. Dual metal sites are bonded to form a metal–metal dimer with a local configuration of N_2_Fe-CuN_2_. Owing to the relatively strong adsorption to NO_3_, the resultant Fe/Cu diatomic catalyst enhances the first rate-determining step, which is used to limit the approach of NO_3_^–^ to the cathode because of its poor affinity with electrodes. Operando DEMS and DFT calculations reveal the reaction pathway and conversion mechanisms from NO_3_^−^ to NH_3_. Compared to Fe/Fe and Cu/Cu configurations, Fe/Cu diatomic sites provide medium interactions toward most other intermediates and lower the overall energy barriers for the conversion from NO_3_^−^ to NH_3_. In brief, Fe/Cu dual sites reconcile the opposite requirements of molecule-catalyst interaction and realize a low energy consumption and high activity synthesis of ammonia. Specifically, the resultant catalyst demonstrates the high activity of ~38.5 mA cm^–2^ (at −0.3 V vs RHE) and selectivity of 92.51% FE for the reduction of NO_3_^–^ to NH_3_. The high NH_3_ yield rate of 1.08 mmol h^−1^ mg^−1^ is achieved at −0.5 V. Overall, this work provides an alternative opportunity for both nitrate abatement and ammonia synthesis and expands the rational design of atomically dispersed catalysts and their applications.

## Methods

### Synthesis of catalyst

GO were prepared via a modified Hummers method^70^. Typically, 3.0 g graphite (Aladdin, 99.9% metals basis) and 18.0 g potassium permanganate (KMnO_4_, Sinopharm Chemical Reagents Co., Ltd., AR, ≥99.5%) were slowly added into the solution of sulfuric acid (H_2_SO_4_, Sinopharm Chemical Reagents Co., Ltd., GR, 95.0–98%, 360 mL) and phosphoric acid (H_3_PO_4_, Sinopharm Chemical Reagents Co., Ltd., GR, ≥85.0%, 40 mL) under stirring. The mixture was heated to 50 °C for 12 h. After cooling down to room temperature by pouring onto ice (~400 mL), a 3.0 mL hydrogen peroxide solution (H_2_O_2_, Aladdin, AR, 30% in H_2_O) was added dropwise. The resultant solid was washed sequentially by de-ionized water, hydrochloric acid (HCl, Sinopharm Chemical Reagents Co., Ltd., AR, 36.0–38.0%), and ethanol (Sinopharm Chemical Reagents Co., Ltd., AR, ≥99.5%). Finally, the GO product was collected by vacuum freeze drying for 24 h. Approximately 100 mg GO was dispersed in an aqueous solution of 200 mL nitric acid (HNO_3_, Sinopharm Chemical Reagents Co., Ltd., GR, 65.0–68.0%). After ultrasonicated for 3 h, the dispersion was centrifuged and the solid phase was cleaned with de-ionized water. Iron chloride hexahydrate (FeCl_3_·6H_2_O, Aladdin, 99%, 9.0 mg), cupric chloride dihydrate (CuCl_2_·2H_2_O, Aladdin, AR, 6.0 mg), and urea (Aladdin, ≥99.5%, 100 mg) were added in the re-dispersed GO suspension (100 mL, ~2.0 mg L^−1^) and then ultrasonicated for 2 h. The mixed suspension was stirred for 12 h and then transferred into a Teflon-lined autoclave. After hydrothermally treated at 180 °C for 12 h, a porous hydrogel was formed. The hydrogel was washed and freeze-dried. The resultant powder was annealed at 800 °C for 2 h at a flowing gas of argon (Ar, Nanjing Special Gas Factory Co., Ltd., 99.999%, 100 sccm) and ammonia (NH_3_, Nanjing Special Gas Factory Co., Ltd., 99.999%, 50 sccm) to yield Fe/Cu-HNG powder.

### Materials characterization

Morphologic and EDS mapping images were collected using a Zeiss Ultra 55 field emission scanning electron microscope. TEM analyses were conducted with an FEI Tecnai G2 20 microscope at 200 kV. Atomic-resolution STEM-HAADF images and EELS spectra were obtained on an FEI Titan G2 60-300 STEM/TEM at 300 kV with a field emission gun or on a JEOL Grand ARM with double spherical aberration correctors. XRD patterns were collected using Rigaku D/MAX 2500 V with Cu Kα radiation (1.5418 Å). XPS analysis was performed on an ESCALab MKII spectrometer with Mg Kα X-ray as the excitation source. Raman spectroscopic characterizations (Renishaw inVia Raman spectroscope) experiments were performed using a 514 nm laser. N_2_ adsorption–desorption isotherms were recorded on an ASAP 2020 accelerated surface area and porosimetry instrument (Micromeritics), equipped with automated surface area. Barrett–Emmett–Teller methods were used to calculate the surface area. The XAS spectra of Fe and Cu K-edge were measured in a fluorescence mode at the beamline BL14W1 of the Shanghai Synchrotron Radiation Facility in China. The concentrations of ions were analyzed by a Shimadzu UV-3600 plus spectrophotometer. The detailed measuring processes are described in detail in the Supplementary Information.

## Supplementary information

Supplementary Information

## Data Availability

All data are available from the authors upon request. Source data are provided with this paper.

## Source data

Source Data
