# Supplementary material for: Fe/Cu diatomic catalysts for electrochemical nitrate reduction to ammonia
Source: Nat Commun. 2023 Jun 19;14:3634. doi: 10.1038/s41467-023-39366-9 (PMC10279643; doi:10.1038/s41467-023-39366-9)
Supplement: Supplementary file 1 — Supplementary Information [file 41467_2023_39366_MOESM1_ESM.pdf]

Supplementary Information

Fe/Cu diatomic catalysts for electrochemical nitrate reduction to ammonia

Shuo Zhang,[1,2,3] Jianghua Wu,[2] Mengting Zheng,[4] Xin Jin,[2] Zihan Shen,[1] Zhonghua Li,[2] Yanjun Wang,[2] Quan Wang,[2] Xuebin Wang,[2] Hui Wei,[2] Jiangwei Zhang,[5] Peng Wang,[2,6] Shanqing Zhang,[4] Liyan Yu,[3] Lifeng Dong,[3] Qingshan Zhu[1,7], Huigang Zhang[1,2,7]* and Jun Lu[8]**

[1] State Key Laboratory of Multiphase Complex Systems, Institute of Process Engineering, Chinese Academy of Sciences, Beijing, 100190, China.

[2] National Laboratory of Solid State Microstructures, Collaborative Innovation Center of Advanced Microstructures, College of Engineering and Applied Sciences, Nanjing University, Nanjing, 210093, China.

[3] College of Materials Science and Engineering, Qingdao University of Science and Technology, Qingdao, 266042, China.

[4] Centre for Clean Environment and Energy and Griffith School of Environment, Griffith University, Gold Coast, Queensland, 4222, Australia.

[5] Dalian National Laboratory for Clean Energy & State Key Laboratory of Catalysis, Dalian Institute of Chemical Physics, Chinese Academy of Sciences (CAS), Dalian, 116023, China.

[6] Department of Physics, University of Warwick, Coventry CV4 7AL, UK.

[7] School of Chemical Engineering, University of the Chinese Academy of Sciences, No. 19(A) Yuquan Road, Shijingshan District, Beijing 100049, PR China.

[8] College of Chemical and Biological Engineering, Zhejiang University, Hangzhou, Zhejiang Province, 310027, China.

**Correspondence and requests for materials should be addressed to J. L or H.Z or Q.Z.*

(e-mail: junzoelu@zju.edu.cn or *hgzhang@ipe.ac.cn* or *qszhu@ipe.ac.cn)*

Contents

Supplementary Methods ········ 1

Synthesis of Homogeneous Catalysts. ········ 1
DEMS Analyses. ········ 1
Detection of NO_3^-. ········ 2
Detection of NO_2^-. ········ 2
Determination of NH_3. ········ 3
^{15}N isotope-labelling experiments by ^{1}H NMR. ········ 3
Faradaic efficiency and yield of NH_3. ········ 4
Energy consumption of NH_3 production. ········ 4
Electrochemical measurements. ········ 5
DFT calculation. ········ 5
Electrochemical nitrate reduction pathway. ········ 6

Supplementary Figures ········ 8

Supplementary Fig. 1 SEM images of rGO and HNG. ········ 8
Supplementary Fig. 2 TEM images of (a) rGO and (b) HNG. ········ 9
Supplementary Fig. 3 HAADF-STEM image of Fe/Cu-HNG. ········ 10
Supplementary Fig. 4 HAADF-STEM image and corresponding EEL spectra. ········ 11
Supplementary Fig. 5 N_2 adsorption-desorption isotherms and the pore-size distribution of (a, b) HNG and (c, d) rGO. ········ 12
Supplementary Fig. 6 XRD patterns of HNG. ········ 13
Supplementary Fig. 7 EDS mapping images of Fe/Cu-HNG. ········ 14
Supplementary Fig. 8 TEM and HRTEM images of Fe/Cu-HNG. ········ 15
Supplementary Fig. 9 Morphology and structure characterization of Fe/Fe-HNG. ········ 16
Supplementary Fig. 10 Morphology and structure characterization of Cu/Cu-HNG. ········ 17
Supplementary Fig. 11 XPS survey of Fe/Cu-HNG, Fe-HNG, and Cu-HNG. ········ 18
Supplementary Fig. 12 Fitting results and WT images of the EXAFS spectra of Cu-foil. ········ 19
Supplementary Fig. 13 Fitting results and WT images of the EXAFS spectra of Fe-foil. ········ 20
Supplementary Fig. 14 Fitting results of the EXAFS spectra of Cu-HNG and Fe-HNG. ········ 21
Supplementary Fig. 15 (a) LSV curves and (b) NH_3 yield rates of Fe/Cu-HNG, Fe/Fe-HNG, Cu/Cu-HNG, and mixture of Fe/Fe-HNG and Cu/Cu-HNG. ········ 22
Supplementary Fig. 16 LSV curves of the Cu-HNG, Fe-HNG, Cu/Cu-HNG, and Fe/Fe-HNG. ········ 23
Supplementary Fig. 17 (a) LSV curves and (b) Fitting curves of the Fe/Cu-HNG. ········ 24
Supplementary Fig. 18 UV-vis calibration curves of NH_3 using ammonium chloride solutions of known concentration as the standard solutions. ········ 25
Supplementary Fig. 19 UV-vis calibration curves of NO_2^- using potassium nitrite solutions of known concentration as the standard solutions. ········ 26
Supplementary Fig. 20 UV-vis calibration curves of NO_3^- using potassium nitrate solutions of known concentration as the standard solutions. ········ 27
Supplementary Fig. 21 (a) Chronoamperometry curve of Fe/Cu-HNG. (b) UV-vis testing curves of Fe/Cu-HNG after different electrolysis times. ········ 28
Supplementary Fig. 22 NH_3 FE of Fe-HNG at varied potentials. ········ 29
Supplementary Fig. 23 NH_3 FE of Cu-HNG at varied potentials. ········ 30
Supplementary Fig. 24 Detection and quantification of $^{15}NH_4^+$ and $^{14}NH_4^+$. ········ 31

Supplementary Fig. 25 FEs of NH_3, NO_2^-, and H_2 generated by Fe/Cu-HNG. ········32
Supplementary Fig. 26 (a) TEM, (b) HRTEM, (c) HAADF-STEM, and (d) Zoomed-in HAADF-STEM images of Fe/Cu-HNG after 24 h test. ········33
Supplementary Fig. 27 The k^3-weighted FT of χ(k)-function from (a) the Cu K-edge EXAFS and (b) the Fe K-edge EXAFS of Fe/Cu-HNG after 24 h test. ········34
Supplementary Fig. 28 LSV curves of the initial and after 24 h test of Fe/Cu-HNG. ········35
Supplementary Fig. 29 (a-f) The NH_3 yield rates and FEs of Fe/Cu-HNG at different nitrate concentrations. ········36
Supplementary Fig. 30 The schematic illustration of customized electrochemically cell. ········37
Supplementary Fig. 31 Full reaction paths for NO_3^- reduction reaction. ········38
Supplementary Fig. 32 The crystallographic model and atomic arrangement for Fe/Cu-HNG. ········39
Supplementary Fig. 33 The structure of (a) Fe single atom and (b) Cu single atom with NO_3* adsorption on the metal atom sites. ········40
Supplementary Fig. 34 The bond length distribution of different Metal site-O on Fe/Cu, Fe/Fe and Cu/Cu diatomic sites, respectively. ········41
Supplementary Fig. 35 Free energy diagram of each intermediate state on the metal atom sites in Fe-Cu, Fe-Fe and Cu-Cu diatomic sites at U =0 V vs. RHE. ········42
Supplementary Fig. 36 Free energy diagram of each intermediate state in Fe-Cu, Fe-Fe and Cu-Cu diatomic sites at U =0.69 V vs. RHE. ········43

Supplementary Tables ········44

Table 1. Elemental composition for the catalysts. ········44
Table 2. EXAFS structure parameters from the different samples. ········45
Table 3. Reported catalysts for the nitrate reduction to ammonia. ········46

Supplementary References ········50

Supplementary Methods

Synthesis of Homogeneous Catalysts. HNG was prepared using the same protocol as heterogeneous catalysts. GO were prepared via a modified Hummers method. GO (100 mg) was dispersed in an aqueous solution of nitric acid (HNO_3, Sinopharm Chemical Reagents Co., Ltd., GR, 65.0~68.0%). After ultrasonicated for 3 h, the dispersion was centrifuged and the solid phase was cleaned with de-ionized water. To prepare Fe/Fe-HNG (or Cu/Cu-HNG), 25 mg iron chloride hexahydrate ($FeCl_3 \cdot 6H_2O$, Aladdin, 99%) or 16 mg copric chloride dihydrate ($CuCl_2 \cdot 2H_2O$, Aladdin, AR), and urea (Aladdin, ≥99.5%, 100 mg) were added in the re-dispersed GO suspension (100 mL, 2 mg L^{-1}) and then ultrasonicated for 2 h. The mixed suspension was stirred for 12 h and then transferred into a Teflon-lined autoclave. After hydrothermally treated at 180 °C for 12 h, a porous hydrogel was formed. The hydrogel was washed and freeze-dried. The resultant powder was annealed at 800 °C for 2 h at a flowing gas of argon (Ar, Nanjing Special Gas Factory Co., Ltd., 99.999%, 100 sccm) and ammonia (NH_3, Nanjing Special Gas Factory Co., Ltd., 99.999%, 50 sccm) to yield homogeneous catalysts. In addition, by lowering the Fe (or Cu) precursor down to 18 mg $FeCl_3$ (or 12 mg $CuCl_2$), we are able to obtain single-atom dominated Fe-HNG (or Cu-HNG) catalysts.

DEMS Analyses. An aqueous solution of 0.1 M KNO_3 (potassium nitrate, Aladdin, AR, 99.0%) and 1 M KOH (potassium hydroxide, Aladdin, GR, 85%) was flowed into a home-made electrochemical cell by a peristaltic pump. Ar gas was bubbled into the electrolyte constantly before and during the DEMS measurements. Fe/Cu-NG coated glassy carbon, Pt wire, and a saturated calomel electrode were used as the working, counter, and reference electrodes, respectively. LSV test was scanned from 0.1 to −0.6 V vs RHE at a rate of 5 mV s^{-1} after the baseline was steady. The mass signals were collected in the process of LSV test. After the end of electrochemical test, waiting for the mass signal to return to baseline, the next cycle started using the same test conditions to minimize errors.

Detection of NO_3^-. The concentration of NO_3^- was determined at different voltages using the ultraviolet-visible (UV–Vis) spectrophotometry. Each voltage was hold for 0.5 h in 1 M KOH and 0.1 M KNO_3 before nitrogen quantification. After that, 1.0 mL electrolyte was removed out of the electrolytic cell and diluted to 5 mL. 0.1 mL HCl (1 M) and 0.01 mL sulfamic acid (Shanghai Macklin Biochemical Technology Co., Ltd., AR, 99.5%, 0.8 wt%) were further added in the solution. After 15 minutes, the UV-vis absorption spectra were recorded with a Shimadzu UV-3600 plus spectrophotometer. The total absorbance of NO_3^- was calculated by the following equation: $A=A_{220}-2*A_{275}$ (where A_{220} and A_{275} are the absorbance coefficients at 220 nm and 275 nm, respectively)[1]. The standard curve can be made by measuring the UV-vis spectra of varied concentrations of KNO_3 solutions.

Detection of NO_2^-. The nitrite concentration was measured by UV–vis spectrophotometry according to the standard method. Firstly, the colour reagent was prepared by mixing sulfonamide (Shanghai Macklin Biochemical Technology Co., Ltd., AR, 99.5%, 4 g), *N*-(1-naphthyl) ethylenediamine dihydrochloride (Shanghai Macklin Biochemical Technology Co., Ltd., >98%, 0.2 g), phosphoric acid (H_3PO_4, Sinopharm Chemical Reagents Co., Ltd., GR, ≥85.0%, 10 mL, ρ=1.685 g mL^{-1}), and deionized water (50 ml). The electrolyte sample should be diluted to the detection range. Then 0.1 mL of the colour reagent was mixed with 5 mL of the sample solution and rested for 20 min at room condition. The absorption intensity at a wavelength of 540 nm was recorded by UV–Vis absorption spectrum. The concentration–absorbance curve was linear fitted using a series of standard KNO_2 (potassium nitrite, Aladdin, AR, 97%) solutions by the same processes. The concentration of NO_2^- product was calculated based on the calibrated curve.

Determination of NH_3. NH_3 was determined by the indophenol blue method according to previous report[2,3]. The electrolyte (1 mL) was transferred into a 5 mL clean vessel. The following solutions, 1 mL NaOH solution (sodium hydroxide, Aladdin, AR, 96%, 1 M) containing salicylic acid (Shanghai Macklin Biochemical Technology Co., Ltd., AR, 99.5%, 5 wt%) and sodium citrate dihydrate (Shanghai Macklin Biochemical Technology Co., Ltd., 99.0%, 5 wt%), 0.5 mL NaClO (sodium hypochlorite solution, Shanghai Macklin Biochemical Technology Co., Ltd., 6~14% active chlorine basis, 0.05 M), and 0.1 mL $Na_2[Fe(NO)(CN)_5]$ (sodium nitroferricyanide(III) dihydrate, Shanghai Macklin Biochemical Technology Co., Ltd., AR, 99.0%, 1 wt%), were added to the vessel sequentially. After 2 h incubation, the UV-vis spectra of the resultant solution were measured from 500 nm to 800 nm. The absorption peak at 655 nm was originated from the formation of indophenol blue. To quantify NH_3 accurately, the concentration-absorbance curve was made by measure a series of standard ammonia chloride (NH_4Cl, Aladdin, GR, 99.8%) with varied concentrations in 0.1 M KOH. The absorbance spectra of a blank sample without NH_3 was subtracted from the measured absorbance of all tested samples for background correction.

^{15}N isotope-labelling experiments by ^1H NMR. ^1H NMR spectroscopy (500 MHz) was used to quantify the $^{15}NH_4^+/^{14}NH_4^+$ yield after electrolysis at −0.3 V (vs RHE). The calibration curves with defined $^{15}NH_4Cl$ (ammonium chloride-^{15}N, Shanghai Macklin Biochemical Technology Co., Ltd., isotopic abundance: 99 atom%, ≥98.5%) concentrations were constructed as standards. In a typical procedure, 125 μl of the standard solution/electrolytes was first diluted to the detection range and adjust to pH 2.0 by adding 0.1 M HCl. Next, the solution was mixed with 0.1 ml DMSO-*d*6 (hexadeuterodimethyl sulfoxide, Shanghai Macklin Biochemical Technology Co., Ltd., D. 99.9%) (include 0.04 wt% $C_4H_4O_4$), where DMSO-*d*6 serves as a solvent and $C_4H_4O_4$ (Maleic acid, Shanghai Macklin Biochemical Technology Co., Ltd., AR, ≥99.0%) as the internal standard. Finally, the prepared solution was tested by a 500MHz NMR spectrometer. The NH_3 product peaks area integral ratio to maleic acid were analyzed to confirm the source of NH_3 qualitatively.

Faradaic efficiency and yield of NH_3. The faradaic efficiency (FE) of NH_3 production was determined by the following equation:

$$FE(NH_3) = (8 \times \mathrm{F} \times C_{NH3} \times \mathrm{V} \times 10^{-16})/(17 \times \mathrm{Q}) \times 100\% \quad (1)$$

Where F is Faraday constant (96485 C mol^{-1}), C_{NH3} is the concentration of NH_3 (μg mL^{-1}) in the electrolyte, V is the volume of the electrolyte, Q is the charge consumed for NH_3 generation.

The yield rate (YR, mg_{NH3} h^{-1} cm^{-2}) of NH_3 can be calculated using the following equation:

$$YR(NH_3) = (C_{NH3} \times \mathrm{V})/(\mathrm{t} \times \mathrm{A}) \times 10^{-3} \quad (2)$$

Where t is the electrolysis time; A is the geometric area of the electrode (1 cm^{-2}).

Energy consumption of NH_3 production. Assuming the overpotential of anodic electrode (the water oxidation) is zero, the half-cell energy efficiency (EE) defined as the ratio of chemical energy to applied electrical power was calculated with the following equation:

$$EE(NH_3) = (1.23 - E_{NH3}^{0}) \times FE(NH_3)/(1.23 - \mathrm{E}) \times 100\% \quad (3)$$

where E_{NH3}^{0} is the equilibrium potential (0.69 V) of nitrate electroreduction to ammonia in alkaline media; $FE(NH_3)$ is the faradaic efficiency for NH_3; 1.23 V is the equilibrium potential of water oxidation (i.e. assuming the overpotential of the water oxidation is zero); E is the applied potential (vs. RHE) for NH_3 production. Energy consumption (EC, Wh g_{NH3}^{-1}) was calculated by $\mathrm{EC} = \frac{\mathrm{P*t}}{\mathrm{m}} = \frac{\mathrm{UI*t}}{\mathrm{m}}$, where U is the voltage (V vs RHE), I is the current density (mA cm^{-2} or mA mg^{-1}), t is the time (h), m is the mass of NH_3 (g).

Electrochemical measurements. Electrochemical properties were measured by a VSP Potentiostat (Bio-Logic Corp., France) in an H-type cell, which has a Nafion membrane (N-117, Dupont) to separate the cathode and anode chambers. Platinum foil and Hg/HgO (1.0 M KOH) were used as the counter and reference electrodes, respectively. The working electrode was prepared using the following procedure. Catalyst powder (4.0 mg), deionized water (1.0 mL), ethanol (1.0 mL), and Nafion solution (Sigma Aldrich, 5 wt%, 50.00 μL) were mixed and sonicated for at least 30 min to form a homogeneous ink. Catalyst ink was drop-casted onto carbon paper (active area: 1×1 cm^{-2}) with a mass loading of 0.240 mg cm^{-2}. The potentials were converted to the reversible hydrogen electrode (RHE) *via* the following equation:

$$E_{(RHE)} = E_{(Hg/HgO)} + 0.059 * pH + 0.098\,V \tag{4}$$

NO_3^- was electrochemically reduced within an electrolyte of 1.0 M KOH and 0.10 M KNO_3, which was first degassed by an ultrapure Ar gas to remove O_2 and N_2. LSV curves were measured at a rate of 5 mV s^{-1} from 0.2 to −0.7 V (vs RHE). Chronopotentiometric tests were carried out at varied potentials to evaluate the faradaic efficiency and yield rate of NH_3. For consecutive recycling tests, the chronopotentiometric tests were performed at −0.3 V (vs RHE) for a prolonged time under stirring. After electrolysis, the electrolyte was analyzed by UV–Vis spectrophotometry.

DFT calculation. The first-principle calculations were performed using the Vienna Ab initio Simulation Package (VASP) code[4,5]. Perdew-Burke-Ernzerhof (PBE) functional within a generalized gradient approximation (GGA) was used to treat the exchange-correlation energy[6]. To describe the expansion of the electronic eigenfunctions, the projector-augmented wave (PAW) method was applied with a kinetic energy cutoff of 500 eV. a 3×3×1 supercell was built for graphene. For structural optimizations, a Γ centered 4×4×1 Monkhorst-Pack k-point sampling was used in the first Brillouin zone, whereas k-point mesh was increased to 8×8×1 for the density of states (DOS) calculations. All atomic positions were fully relaxed until energy and force reached the tolerance of 1×10^{-5} eV and 0.02 eV $Å^{-1}$, respectively.

The vacuum thickness was set to be 15 Å to minimize interlayer interactions. The solvation effect was not included since the ignorable energy change was witnessed. VASPKIT was adopted to obtain the DOS diagrams[7]. Wannier orbitals were calculated using a wannier90 code[8].

Electrochemical nitrate reduction pathway. Based on computational hydrogen electrode (CHE) model[9], the Gibbs free energy (ΔG) calculations of each elementary step can be determined as:

$$\Delta G = \Delta E + \Delta E_{ZPE} - T\Delta S + \Delta U + \Delta pH \quad (5)$$

where ΔE is the energy obtained from DFT calculations. ΔE_{ZPE} and ΔS are the correction of zero-point energy and entropy, respectively. T is temperature (298.15 K). ΔU and ΔpH represent the effect of voltage and pH, respectively.

To avoid calculate the free energy of charged NO_3^- directly, gaseous HNO_3 is chosen as a reference instead[10]. Following the method of previous report, the adsorption energy of NO_3^- (ΔG^*_{NO3}) could be determined by:

$$\Delta G^*_{NO3} = G^*_{NO3} - G^* - G_{HNO3(g)} + 1/2 G_{H2(g)} + G_{correct} \quad (6)$$

where G^*_{NO3}, G^*, $G_{HNO3(g)}$, and $G_{H2(g)}$ are the Gibbs free energy of NO_3 adsorbed on substrate, HNO_3, and H_2 molecules in the gas phase, respectively. $\Delta G_{correct}$ denotes the correction of adsorption energy and is set to 0.392 eV. Electrochemical reduction from nitrate to NH_3 involves nine protons and eight electrons. The whole reaction can be summarized as:

$$NO_3^- + 9H^+ + 8e^- \rightarrow NH_3 + 3H_2O$$

The elementary steps of reduction pathway on catalyst were simulated according to the following reactions:

$$* + NO_3^- \rightarrow NO_3^* + e^-$$

$$NO_3^* + H^+ + e^- \rightarrow NO_3^*H$$

$$NO_3^*H + H^+ + e^- \rightarrow NO_2^* + H_2O$$

$$NO_2^* + H^+ + e^- \rightarrow NO_2^*H$$

$$NO_2^*H + H^+ + e^- \rightarrow NO^* + H_2O$$

$$NO^* + H^+ + e^- \rightarrow NOH^*$$

$$NOH^* + H^+ + e^- \rightarrow HNOH^*$$

$$HNOH^* + H^+ + e^- \rightarrow H_2NOH^*$$

$$H_2NOH^* + H^+ + e^- \rightarrow NH_2^* + H_2O$$

$$NH_2^* + H^+ + e^- \rightarrow NH_3^*$$

$$NH_3^* \rightarrow * + NH_3$$

where * represents the adsorption site.

Supplementary Figures

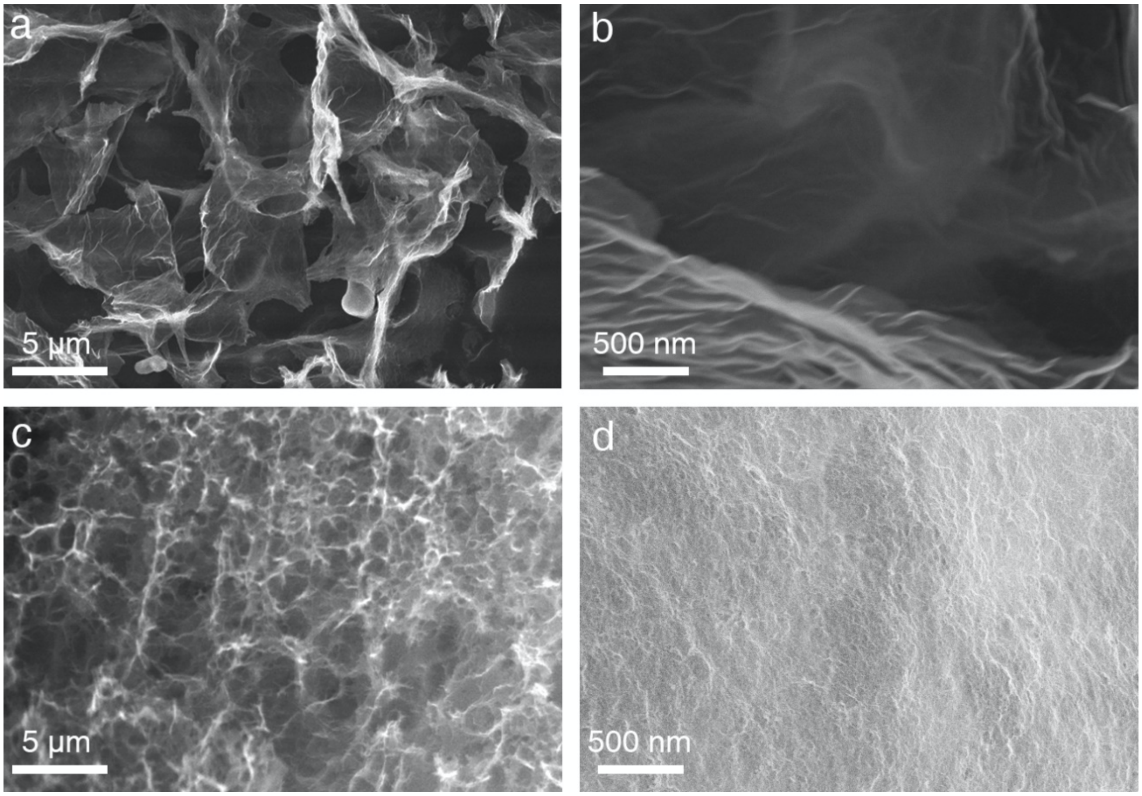

Supplementary Fig. 1 SEM images of (a, b) rGO and (c, d) HNG indicate that HNG has an interconnected vesicle-like structure.

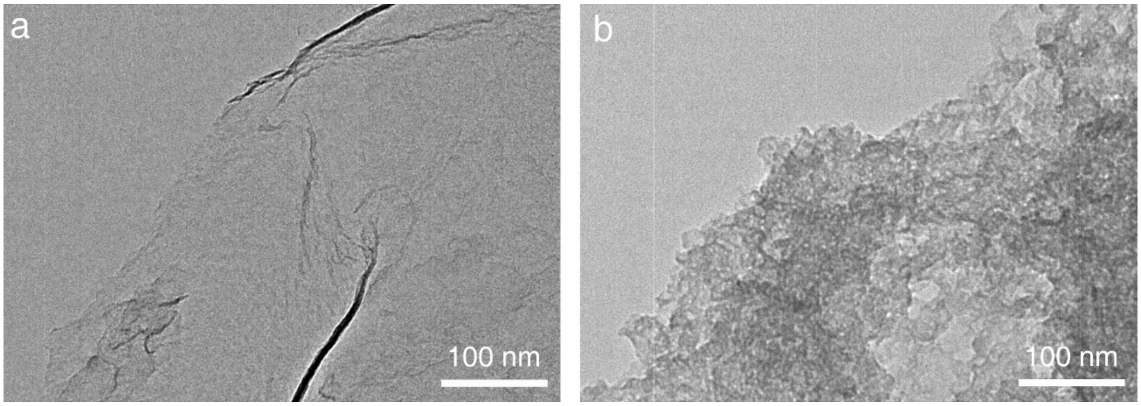

Supplementary Fig. 2 TEM images of (a) rGO and (b) HNG.

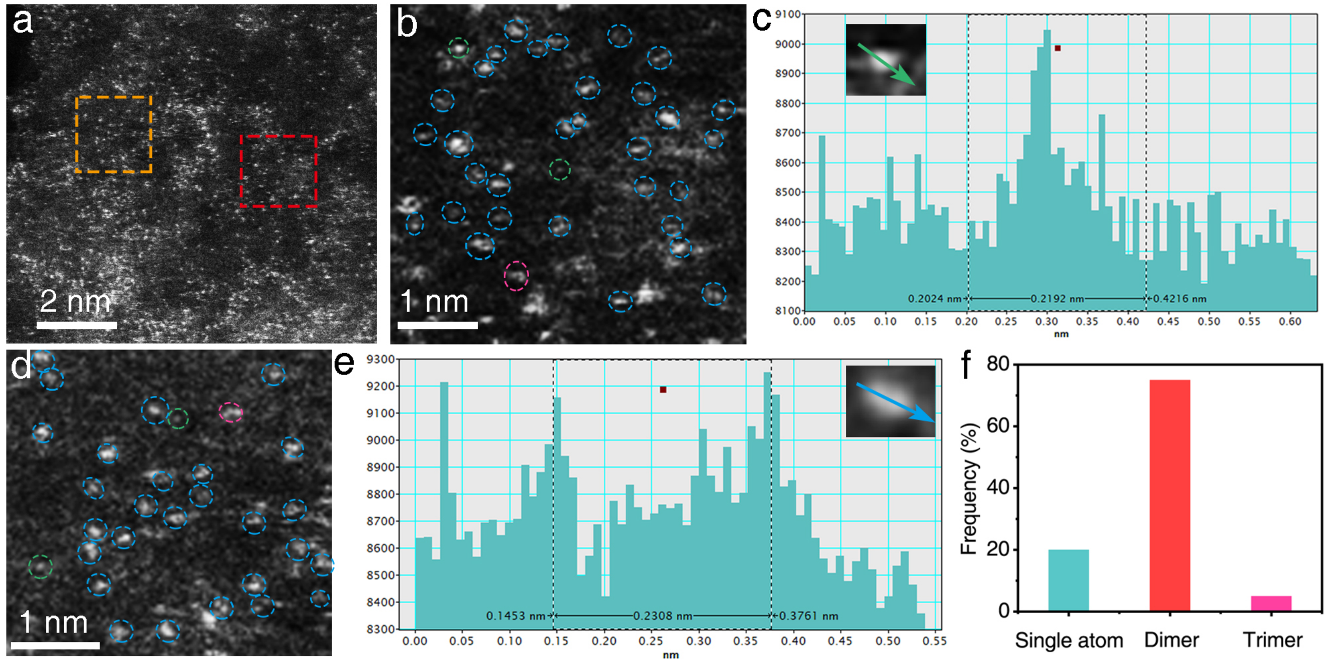

Supplementary Fig. 3 (a) HAADF-STEM image of Fe/Cu-HNG, (b) Magnified HAADF-STEM image of the red rectangular area. (c) Intensity profiles of the single atom site in b. (d) Magnified HAADF-STEM image of the orange rectangular area. (e) Intensity profiles of the diatomic pair in d. (f) Statistical distribution analysis of single-atoms, dimmers, and trimmers in Fe/Cu-HNG. The HAAFS-STEM images shows dimmer combination. However, there still exists a possibility that two adjacent atoms in two stacking graphene layers are imaged and mistaken as diatomic sites. Therefore, a single layer of holey graphene (see Supplementary Fig. 7) should be chosen to take the HAADF-STEM images in order to avoid the overlay imaging of two isolated single atoms. The XRD pattern in Fig. 2i shows a peak around 25.8 ^{o}, which corresponds to the interlayer distance of 3.47 Å between multi-layer graphene. The EXAFS fitting data show the second shell peak (metal-metal path) around 2.25 Å, which excludes the possibility of dual atoms in the different layers. By analyzing the STEM images, the percentage of diatomic pairs accounted for ~75.43%.

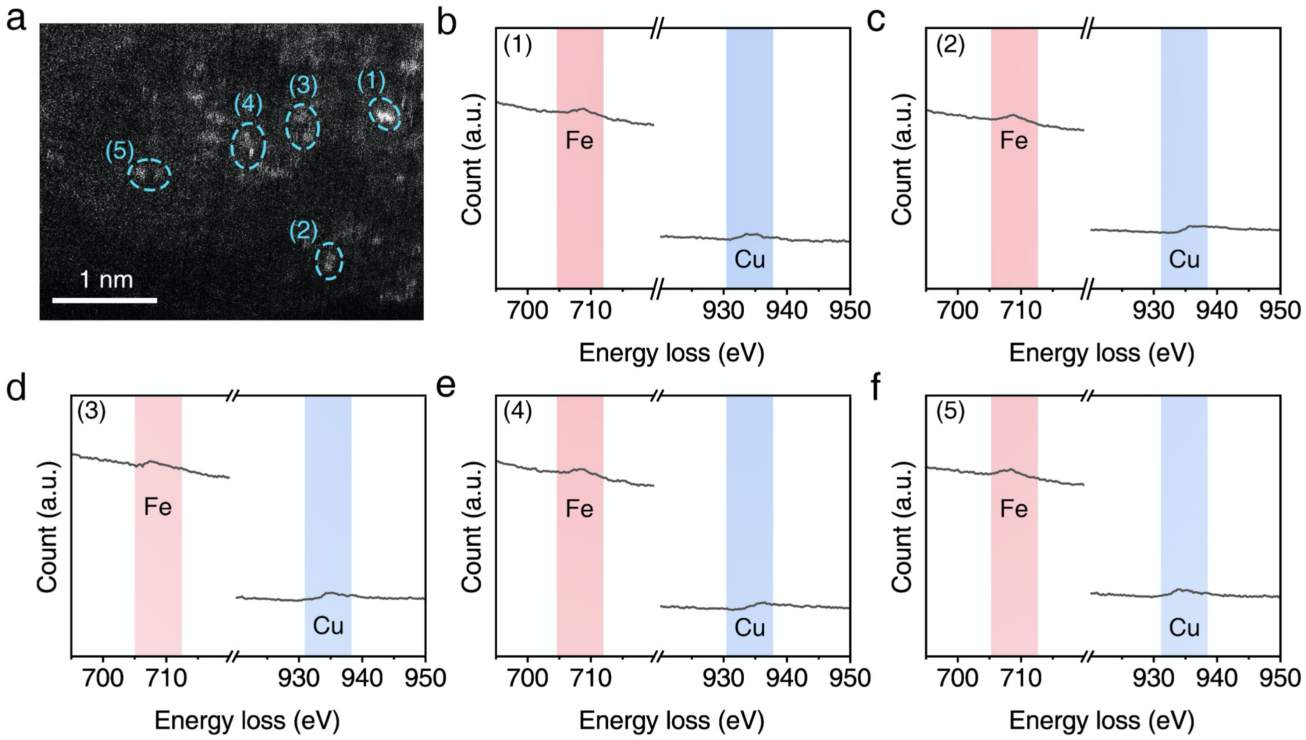

Supplementary Fig. 4 (a) HAADF-STEM image and (b-f) corresponding EEL spectra taken at the different sites. The Fe and Cu signals in the EELS spectra affirmatively indicates the dual Fe-Cu sites in Fe/Cu-HNG.

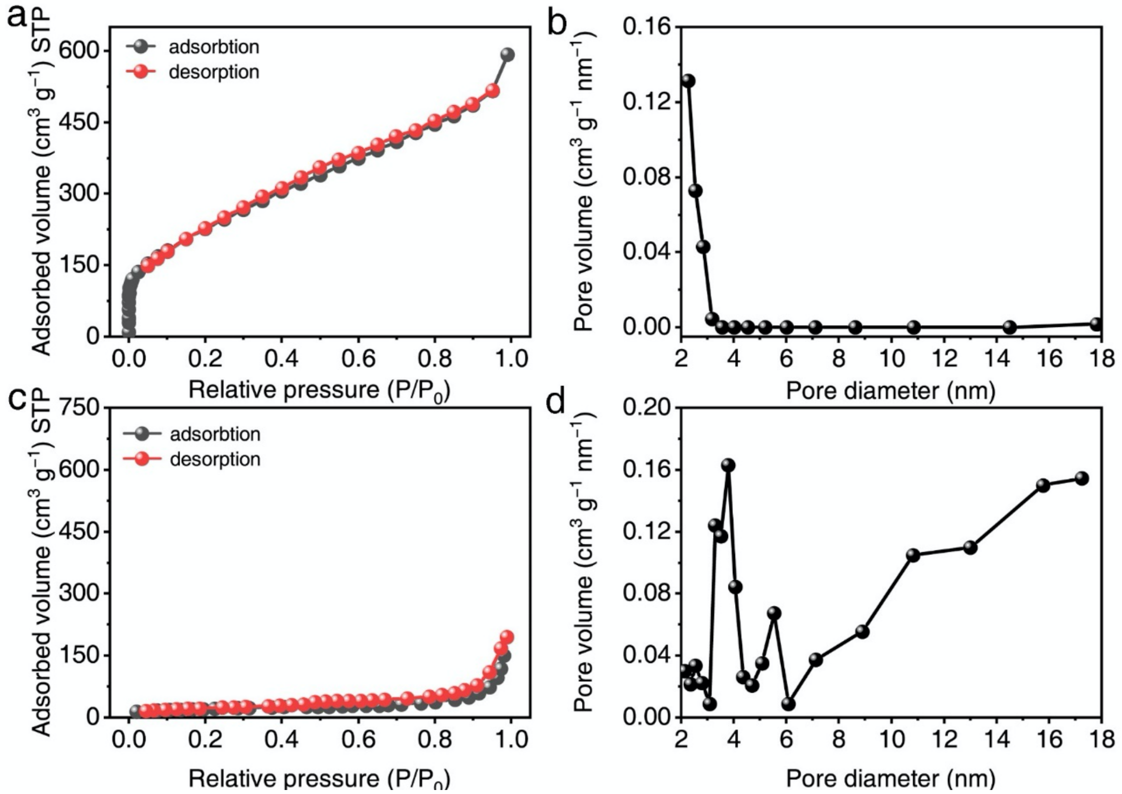

Supplementary Fig. 5 N_2 adsorption-desorption isotherms and the pore-size distribution of (a, b) HNG and (c, d) rGO.

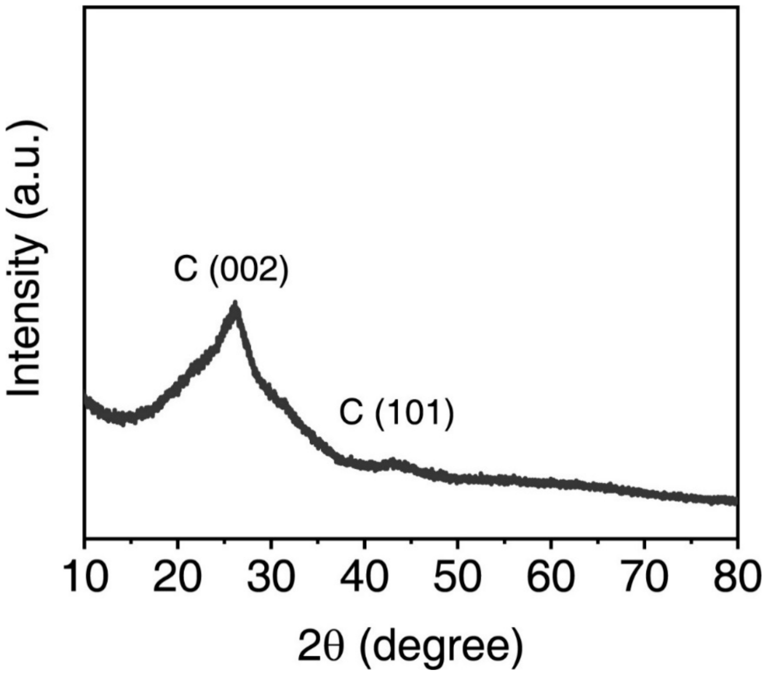

Supplementary Fig. 6 XRD patterns of HNG. Two peaks at ~26° and 43° are attributed to the (002) and (101) planes of graphitic carbon. As compared to Fig. 2i, it could be concluded that no crystalline impurities are observed after loading Fe/Cu.

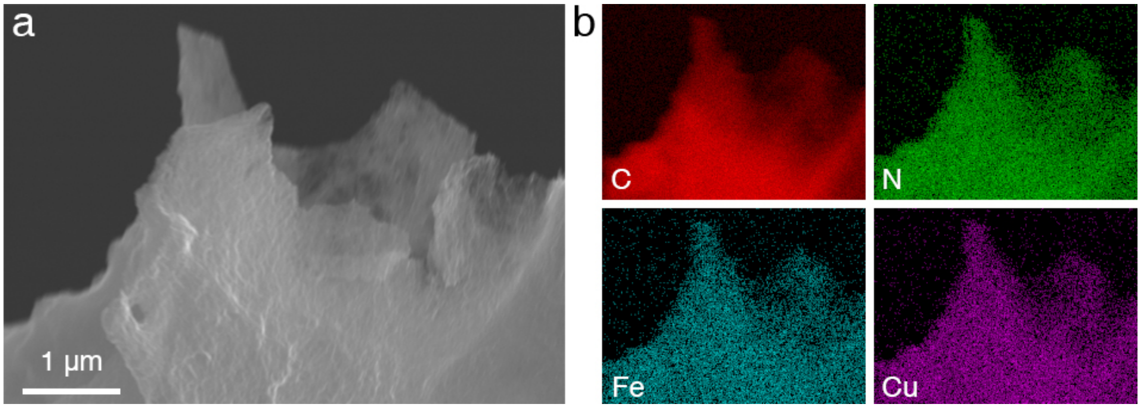

Supplementary Fig. 7 (a) SEM and (b) EDS mapping images of Fe/Cu-HNG indicate the existence of Fe, Cu, N, and C elements throughout the graphene layer.

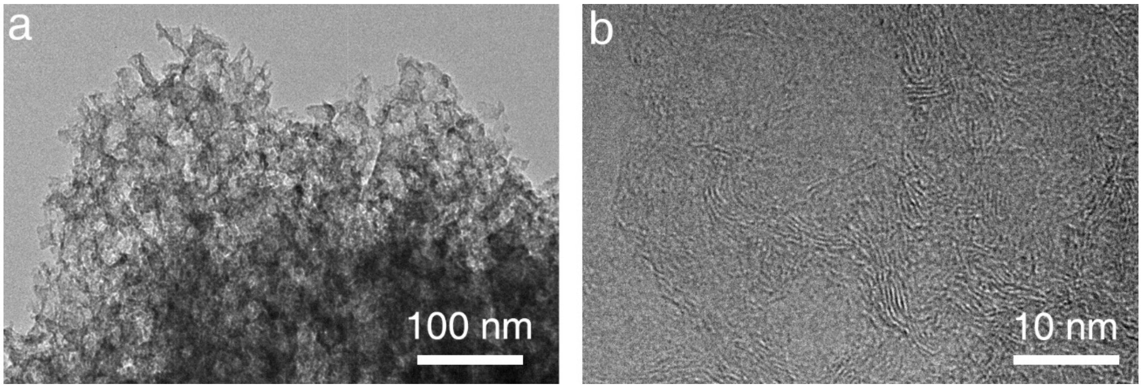

Supplementary Fig. 8 (a) TEM and (b) HRTEM images of Fe/Cu-HNG show randomly orientated graphitic layers without obvious metal particles.

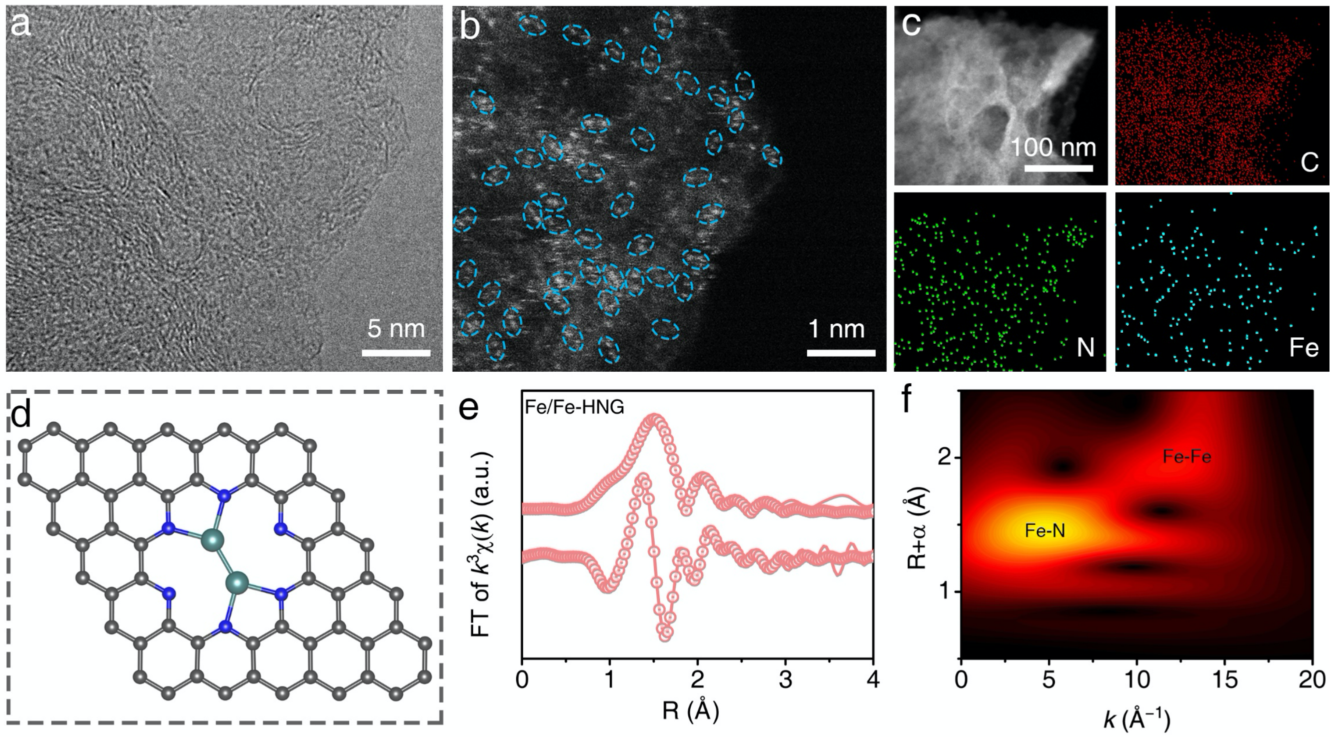

Supplementary Fig. 9 (a) HRTEM and (b) HADDF-STEM images of Fe/Fe-HNG show randomly orientated graphitic layers without obvious metal nanoparticles. (c) STEM and EDX elemental mapping images of C, N, and Fe in Fe/Fe-HNG. (d) Proposed geometric model of Fe/Fe-HNG (The calculated bond length of Fe-N is ~1.92 Å, Fe-Fe is ~2.15 Å). (e) Fitting results of the EXAFS spectra of Fe/Fe-HNG in the R space of Fe K-edge. (f) WT image of the Fe K-edge from Fe/Fe-HNG.

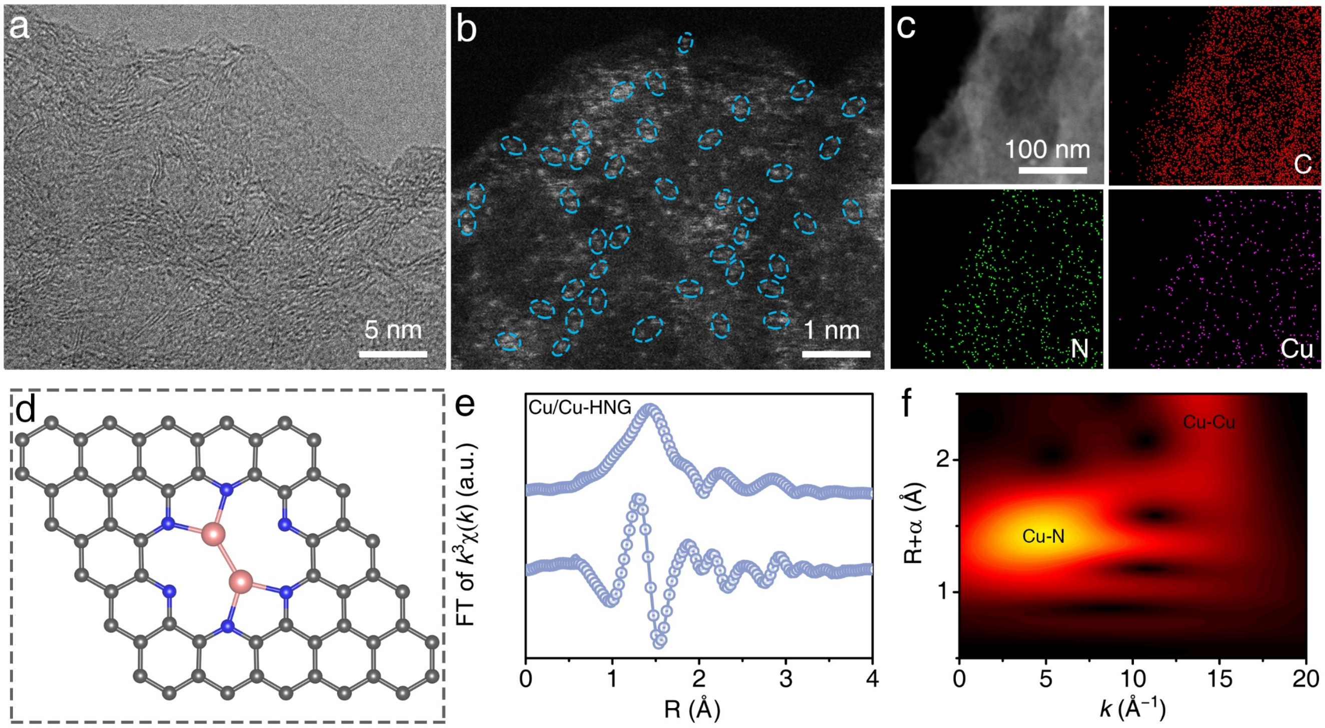

Supplementary Fig. 10 (a) HRTEM and (b) HADDF-STEM images of Cu/Cu-HNG show randomly orientated graphitic layers without obvious metal particles. (c) EDX elemental mapping images of C, N, and Cu in Cu/Cu-HNG. (d) Proposed geometric model of Cu/Cu-HNG (The calculated bond length of Cu-N is ~1.95 Å, Cu-Cu is ~2.35 Å). (e) Fitting results of the EXAFS spectra of Cu/Cu-HNG in R space of Cu K-edge. (f) WT image of the Cu K-edge from Cu/Cu-HNG.

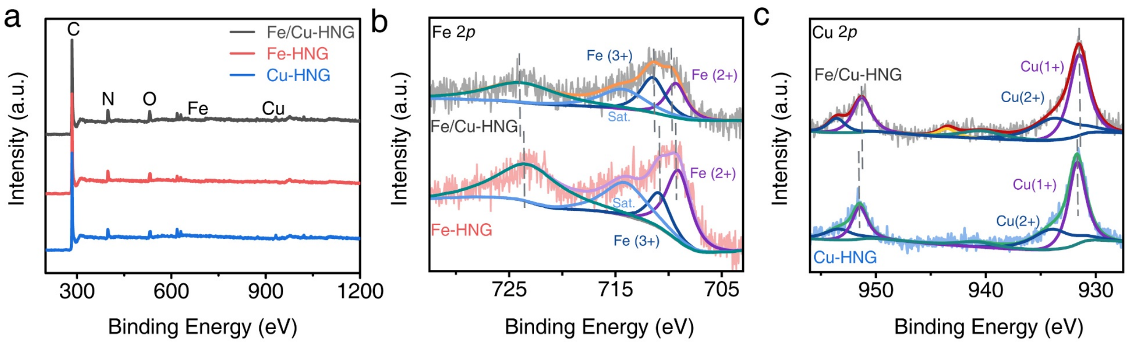

Supplementary Fig. 11 (a) XPS survey of Fe/Cu-HNG, Fe-HNG, and Cu-HNG. High resolution XPS of (b) Fe 2*p* spectra in Fe/Cu-HNG and Fe-HNG, (c) Cu 2*p* spectra in Fe/Cu-HNG and Cu-HNG.

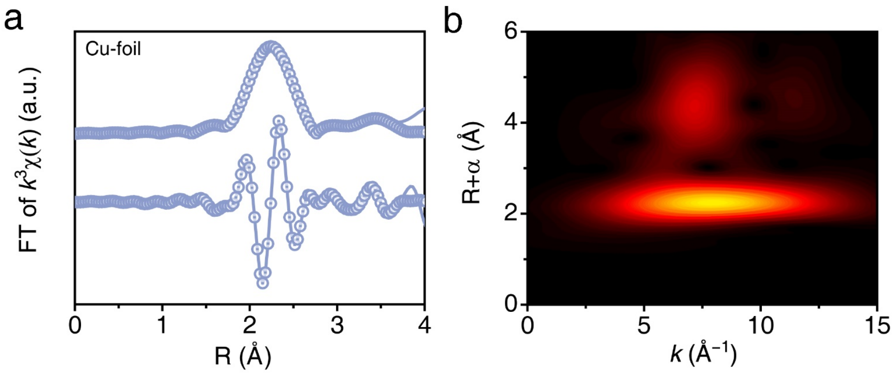

Supplementary Fig. 12 (a) Fitting results of the EXAFS spectra of Cu-foil in R space of Cu K-edge. (b) WT images of the Cu K-edge from Cu-foil.

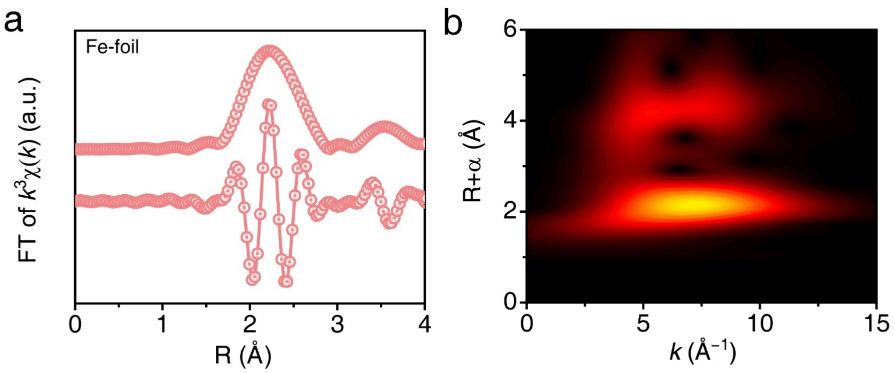

Supplementary Fig. 13 (a) Fitting results of the EXAFS spectra of Fe-foil in R space of Fe K-edge. (b) WT images of the Fe K-edge from Fe-foil.

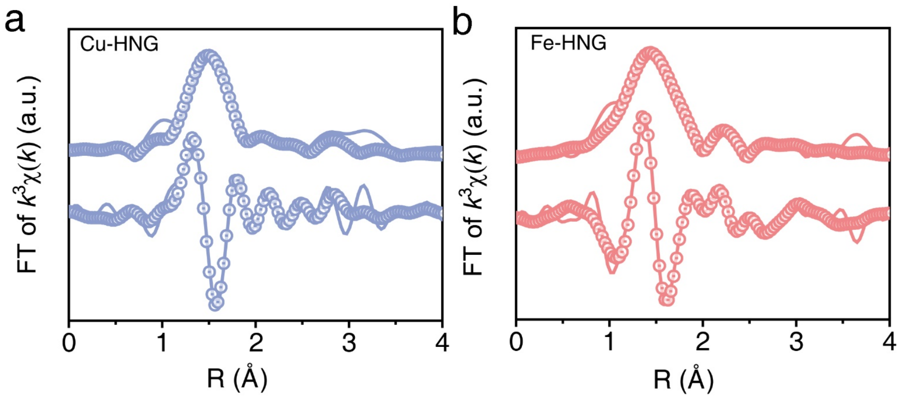

Supplementary Fig. 14 (a) Fitting results of the EXAFS spectra of Cu-HNG and (b) Fe-HNG at R space.

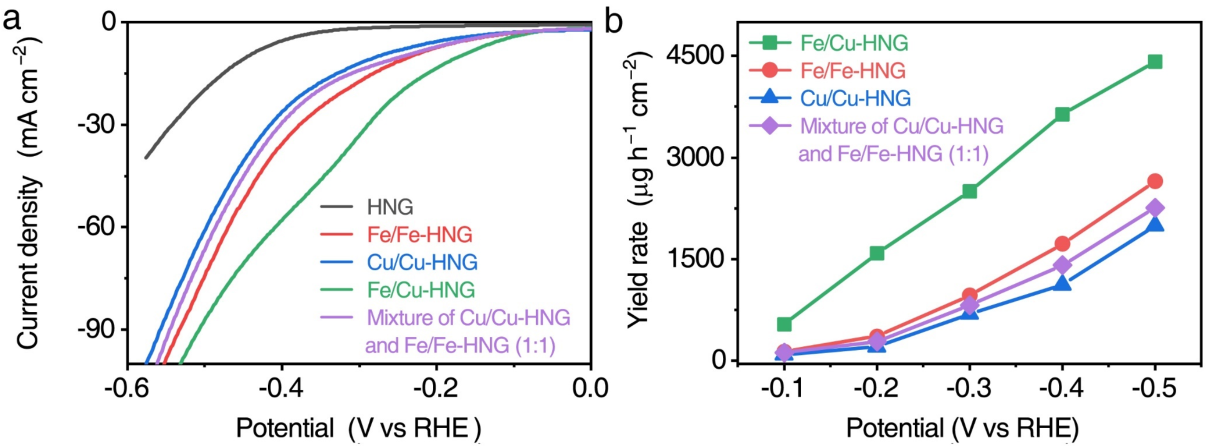

Supplementary Fig. 15 (a) LSV curves of Fe/Cu-HNG, Fe/Fe-HNG, Cu/Cu-HNG, and mixture of Fe/Fe-HNG and Cu/Cu-HNG (mass ratio 1:1) in 1 M KOH and 0.1 M KNO_3. (b) NH_3 yield rates of Fe/Cu-HNG, Fe/Fe-HNG, Cu/Cu-HNG, and mixture of Fe/Fe-HNG and Cu/Cu-HNG (1:1).

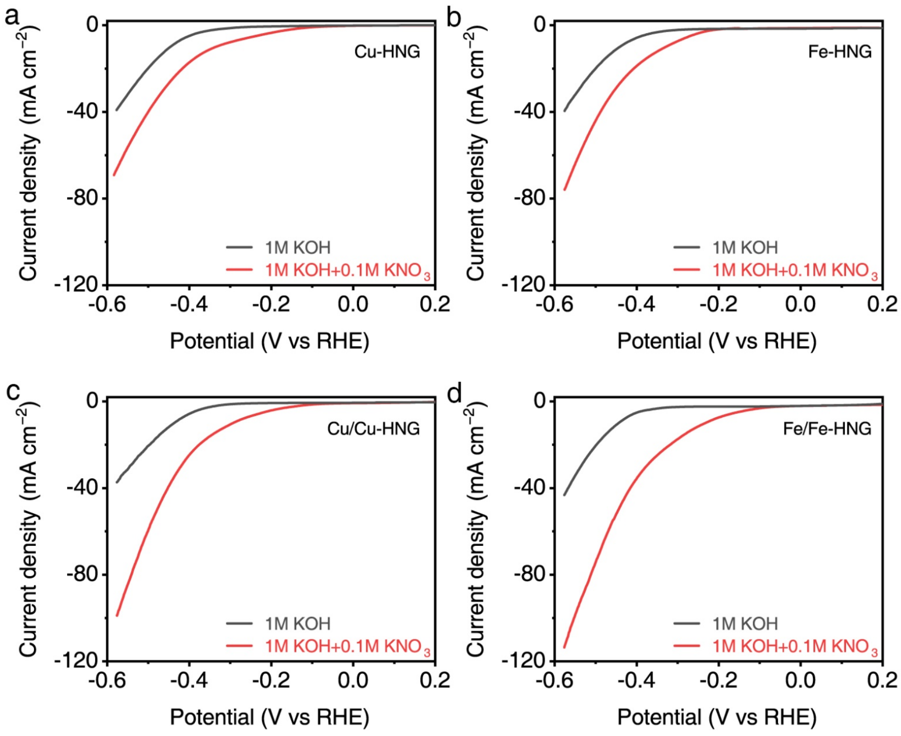

Supplementary Fig. 16 LSV curves of the (a) Cu-HNG, (b) Fe-HNG, (c) Cu/Cu-HNG, and (d) Fe/Fe-HNG in 1 M KOH and 1 M KOH with 0.1 M KNO_3 electrolyte.

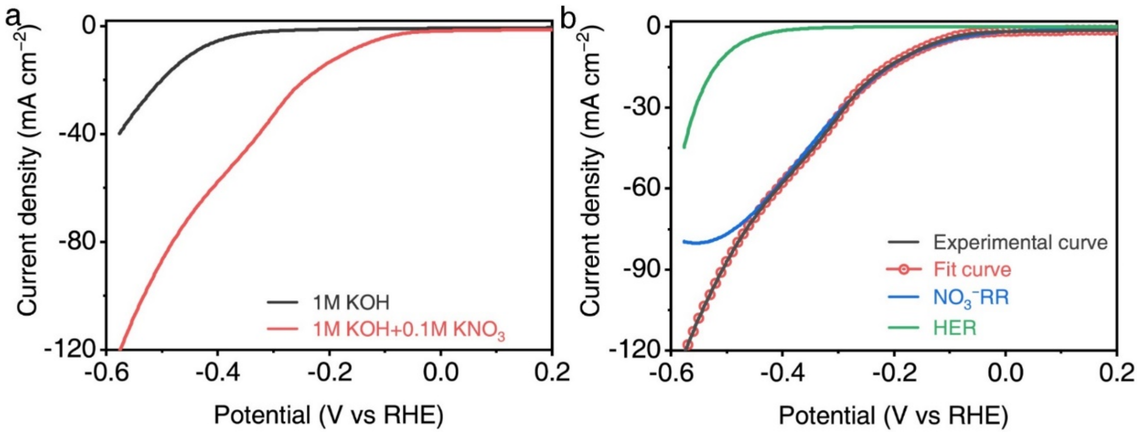

Supplementary Fig. 17 (a) LSV curves of the Fe/Cu-HNG in 1 M KOH and 1M KOH with 0.1M KNO_3 electrolyte. (b) Fitting curves of HER and NO_3^-RR parts of LSV curve of Fe/Cu-HNG. The NO_3^-RR accounts for 85.7% of the total reaction. The transferred electrons of individual reactions were calculated by integrating the current curves of the NO_3^-RR or HER with respect to time.

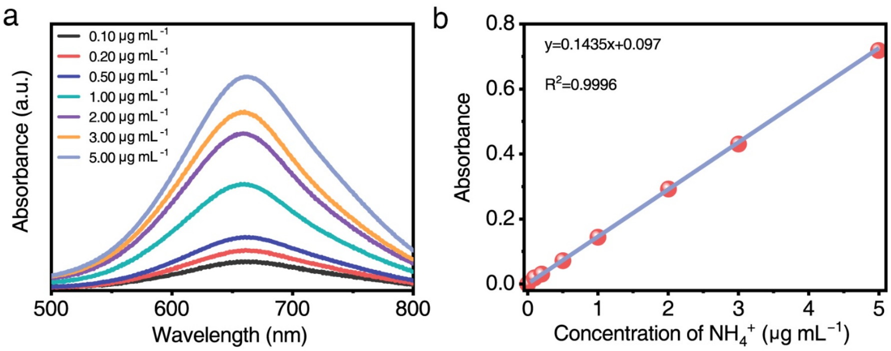

Supplementary Fig. 18 UV-vis calibration curves of NH_3 using ammonium chloride solutions of known concentration as the standard solutions. (a) UV-vis curves of indophenol assays with NH_4^+ and (b) calibration curve at 655 nm for different NH_4^+ concentrations.

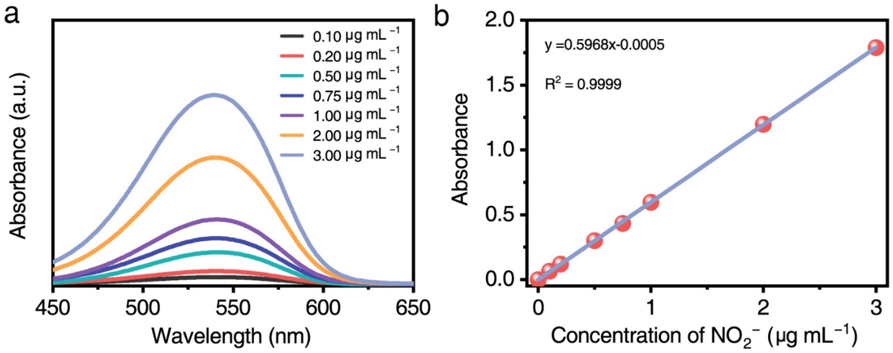

Supplementary Fig. 19 UV-vis calibration curves of NO_2^- using potassium nitrite solutions of known concentration as the standard solutions. (a) UV-vis curves and (b) calibration curve at 540 nm for different NO_2^- concentrations.

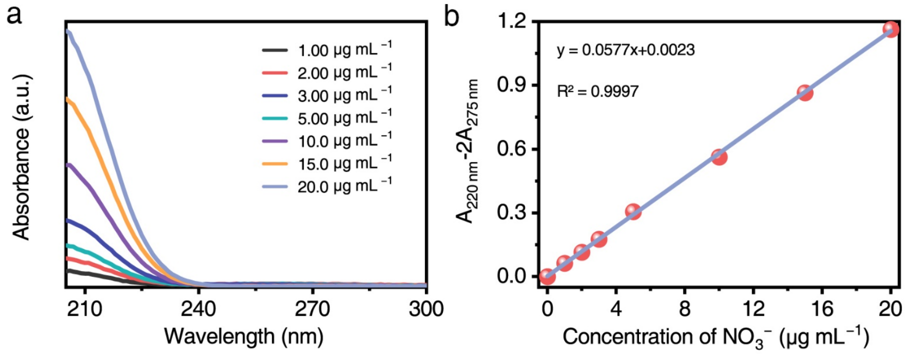

Supplementary Fig. 20 UV-vis calibration curves of NO_3^- using potassium nitrate solutions of known concentration as the standard solutions. (a) UV-vis curves and (b) calibration curve for different NO_3^- concentrations.

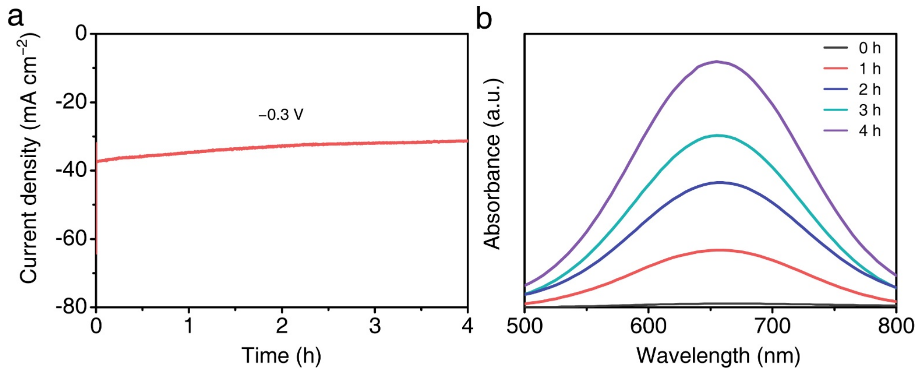

Supplementary Fig. 21 (a) Chronoamperometry curve of Fe/Cu-HNG at −0.3 V (vs RHE) for 4 h. (b) UV-vis testing curves of Fe/Cu-HNG after different electrolysis times.

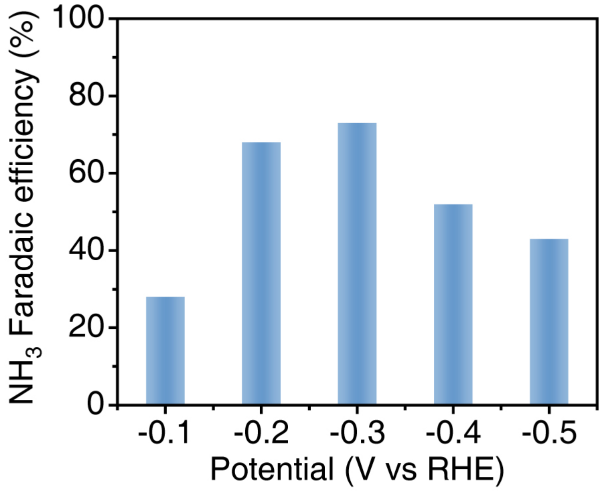

Supplementary Fig. 22 NH_3 FE of Fe-HNG at varied potentials.

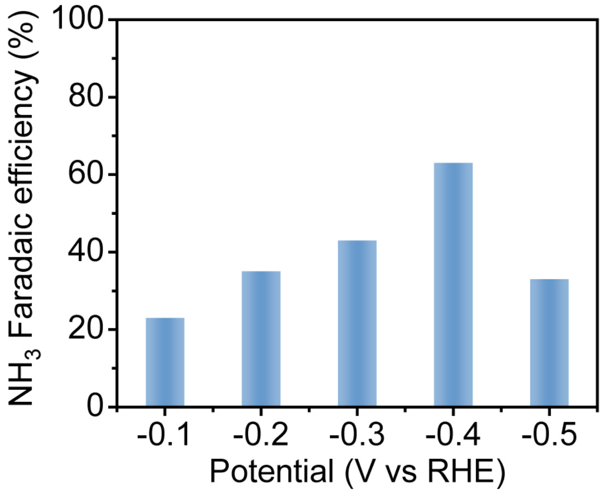

Supplementary Fig. 23 NH_3 FE of Cu-HNG at varied potentials.

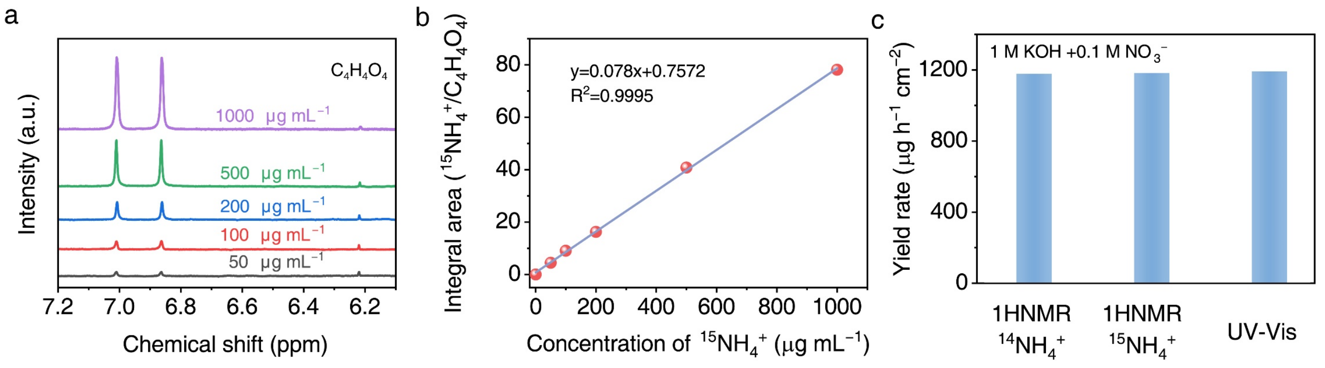

Supplementary Fig. 24 Detection and quantification of $^{15}NH_4^+$ and $^{14}NH_4^+$ by ^1H NMR spectra. (a) ^1H NMR spectra of $^{15}NH_4^+$ ions at different concentrations. $C_4H_4O_4$ with a constant concentration was used as an external standard (with the proton signal at d = 6.25 ppm). (b) Calibration curve for $^{15}NH_4^+$ detection using ^1H NMR, where $^{15}NH_4^+$ peak area integrals were normalized to that of $C_4H_4O_4$. The normalized peak area integral of $^{15}NH_4^+$ is positively correlated to the concentrations of $^{15}NH_4^+$. (c) Comparison of the ammonia yield rate over Fe/Cu-HNG quantified by the ^1H NMR and UV-vis spectra.

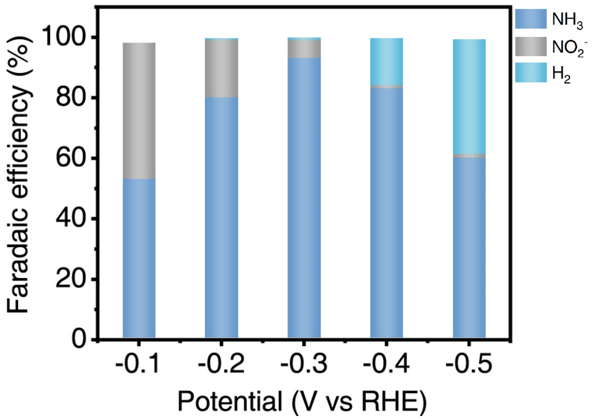

Supplementary Fig. 25 FEs of NH_3, NO_2^-, and H_2 generated by Fe/Cu-HNG at varied potentials. Given that their total efficiencies were maintained around 98~99%, only NH_3, NO_2^-, and H_2 were measured.

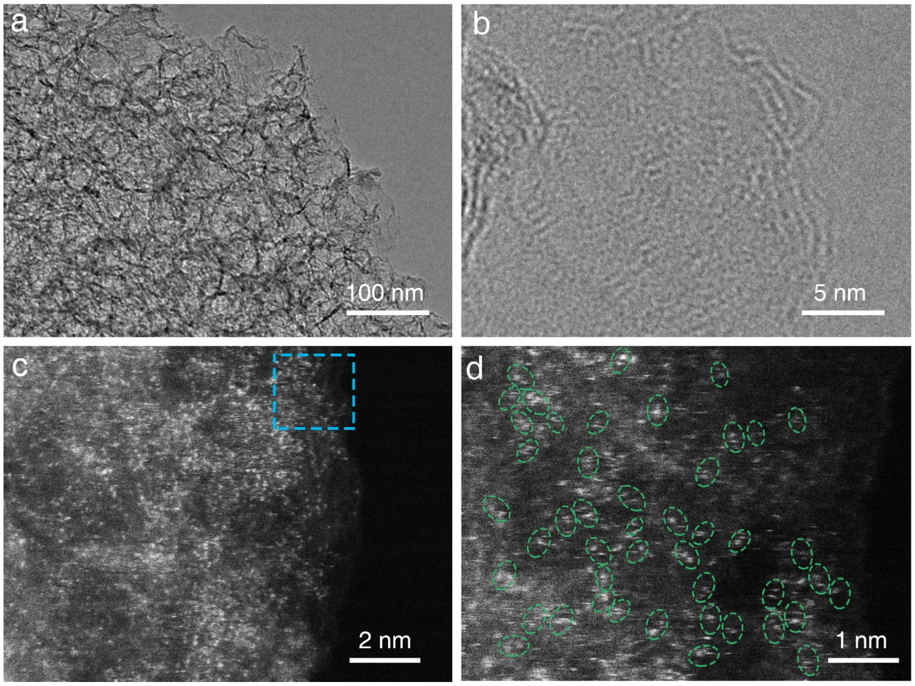

Supplementary Fig. 26 (a) TEM, (b) HRTEM, (c) HAADF-STEM, and (d) Zoomed-in HAADF-STEM (dual-atoms sites were marked with green dashed circles) images of Fe/Cu-HNG after 24 h test.

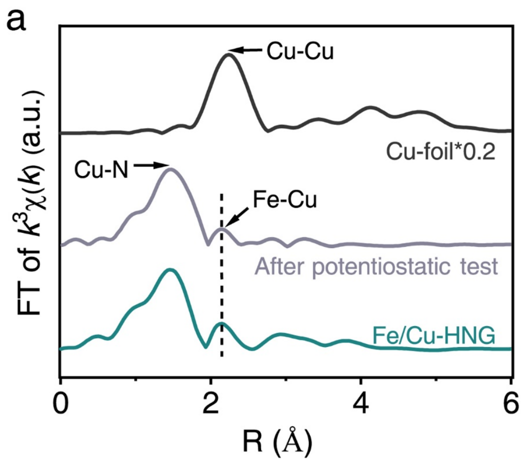

Supplementary Fig. 27 The k^3-weighted FT of χ(k)-function from (a) the Cu K-edge EXAFS and (b) the Fe K-edge EXAFS of Fe/Cu-HNG after 24 h test.

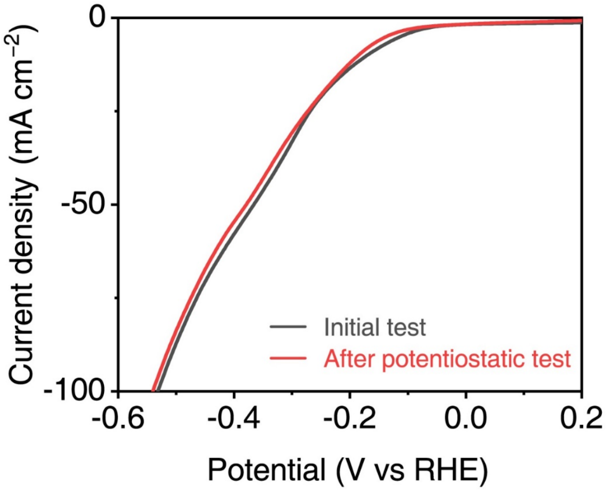

Supplementary Fig. 28 LSV curves of the initial and after 24 h test of Fe/Cu-HNG.

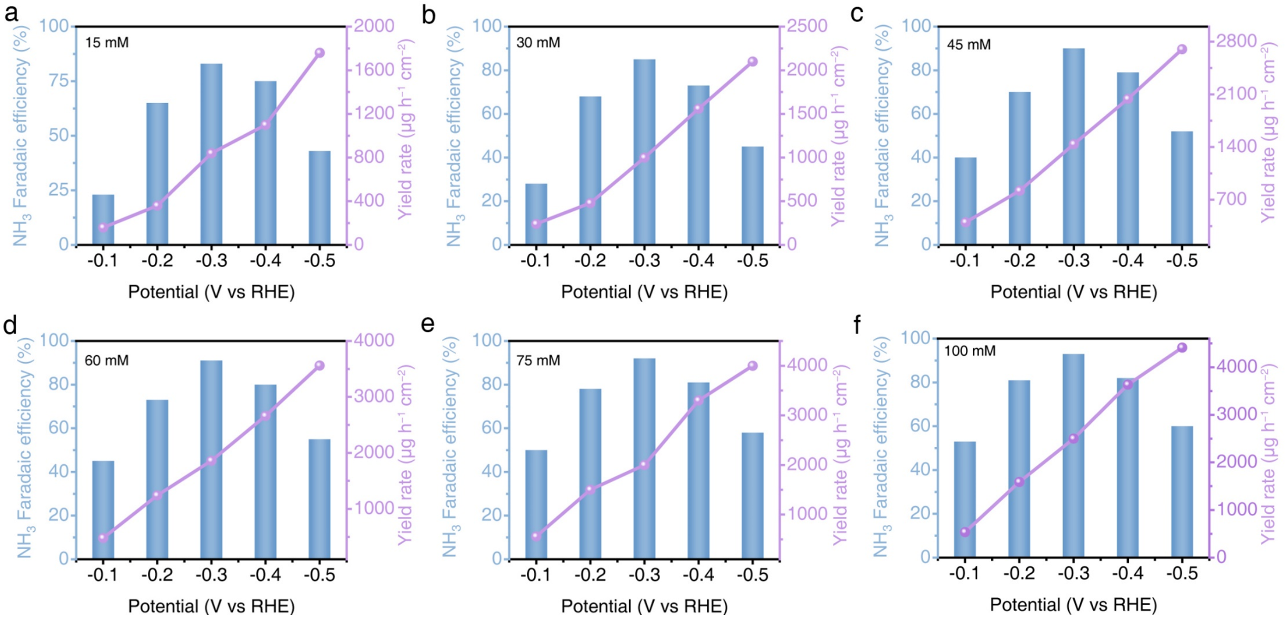

Supplementary Fig. 29 (a-f) The NH_3 yield rates and FEs of Fe/Cu-HNG at different nitrate concentrations. The catalytic performance of Fe/Cu-HNG was examined at different NO_3^- concentrations range from 15 mM to 100 mM because the wide concentration range of various nitrate concentrations may exist in different water sources. The maximal FEs of NO_3^- to NH_3 in the tested concentration range were 83~93% at −0.3 V (vs RHE), respectively. The low YEs in the low nitrate concentration solution result from the low diffusion flux of nitrate from the electrolyte to the catalyst surface. Furthermore, the FEs decreased dramatically at the more negative potential owing to the competitive HER. In general, the catalyst presented the appreciable ammonia yield rates and high appreciable selectivity under different nitrate concentrations, demonstrating the high activity of Fe/Cu-HNG.

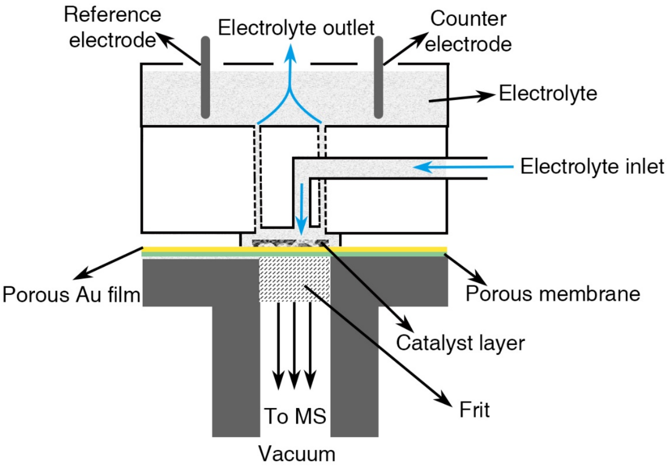

Supplementary Fig. 30 The schematic illustration of customized electrochemically cell.

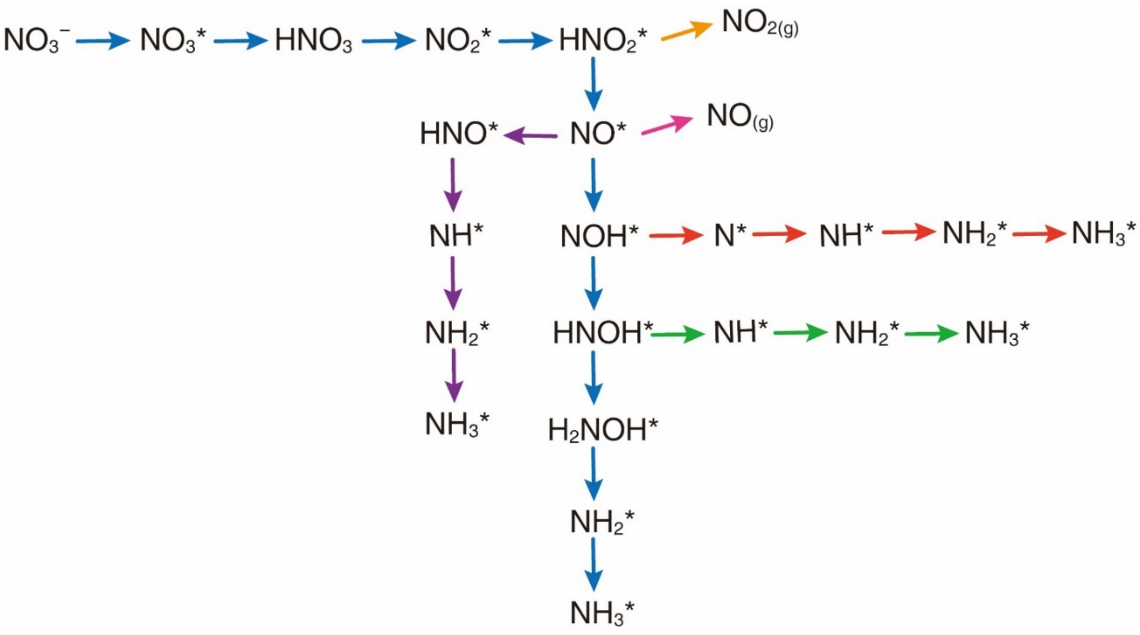

Supplementary Fig. 31 Full reaction paths for NO_3^- reduction reaction.

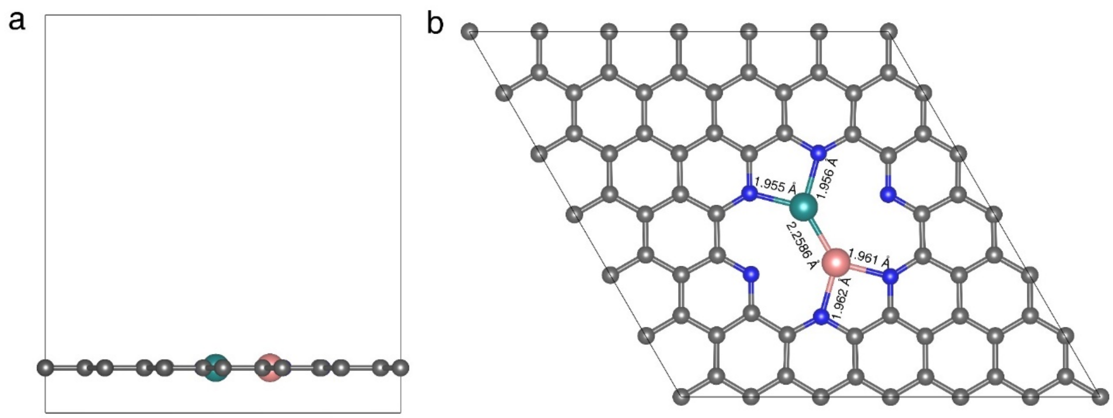

Supplementary Fig. 32 (a, b) The crystallographic model and atomic arrangement for Fe/Cu-HNG. After optimization, the distance between two adjacent Fe and Cu atoms is around 2.26 Å, which is close to the observed separation from the STEM image in Fig. 2c.

In the local structure of N_2Fe-CuN_2, each metal atom is triply coordinated with two nitrogen atoms and one metal (similar to a Y-type ML_3 coordination). The $4sp^2$ hybrid orbitals of Fe form three in-plane σ bonds. The Fe $\boldsymbol{3d_{xy}}$ orbital with some $\boldsymbol{4p_x}$ mixing and $\boldsymbol{3d_{x^2-y^2}}$ orbital with some $\boldsymbol{4p_y}$ mixing could also contribute to the bonding interaction with 2 N and Cu while the Fe $\boldsymbol{3d_{z^2}}$ orbital remains weakly bonding with them. The Fe $\boldsymbol{3d_{yz}}$ and $\boldsymbol{3d_{xz}}$ orbitals weakly bond with 2 N $\boldsymbol{2p_z}$, and may also form weak d-d π and d-d δ interactions with Cu, respectively.

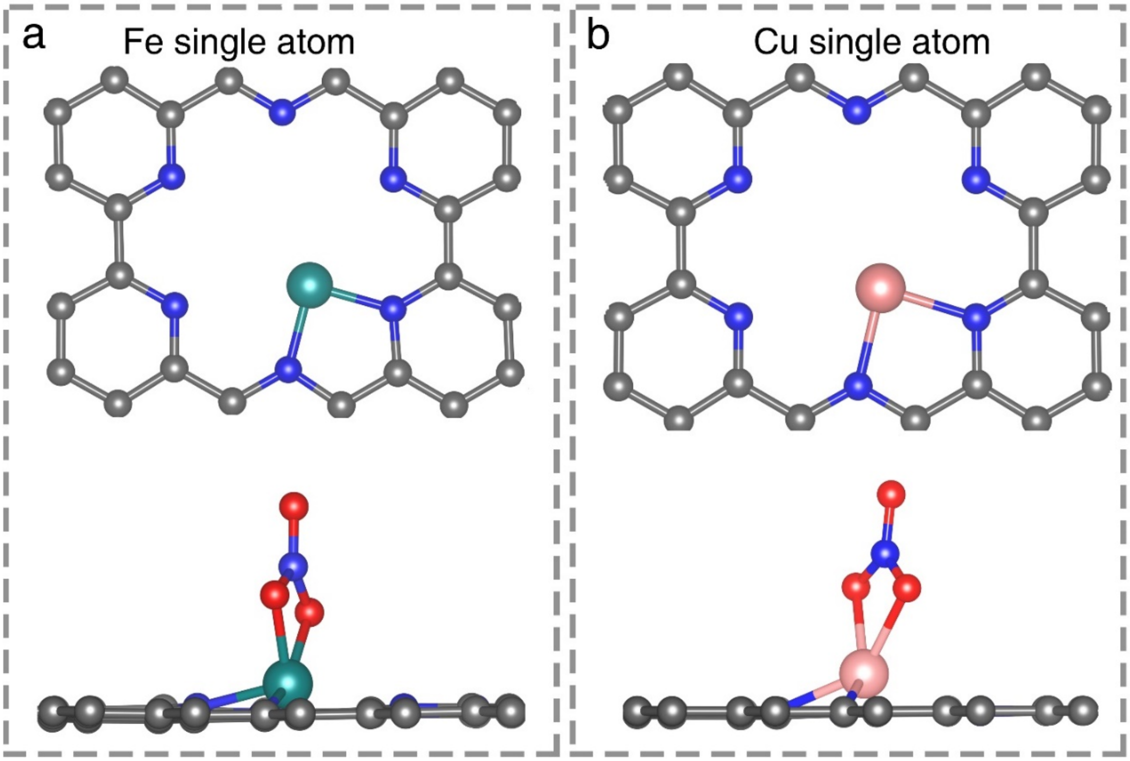

Supplementary Fig. 33 The structure of (a) Fe single atom and (b) Cu single atom with NO_3* adsorption on the metal atom sites.

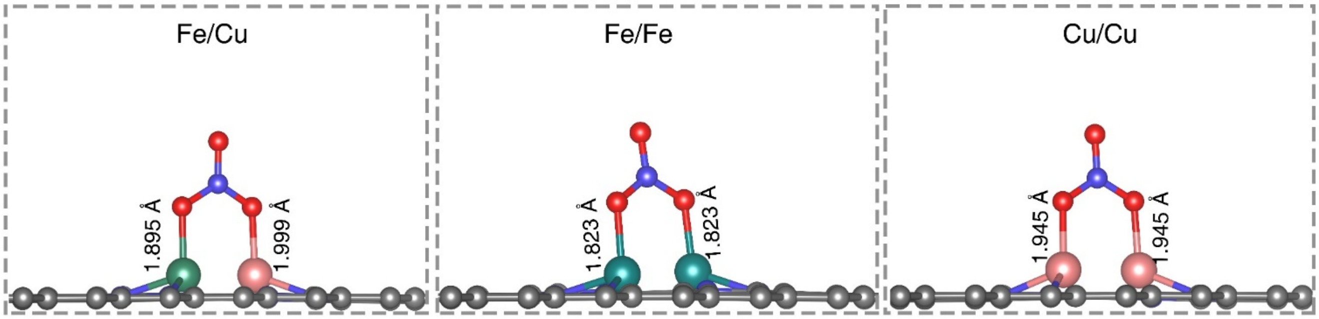

Supplementary Fig. 34 The bond length distribution of different Metal site-O on Fe/Cu, Fe/Fe and Cu/Cu diatomic sites, respectively.

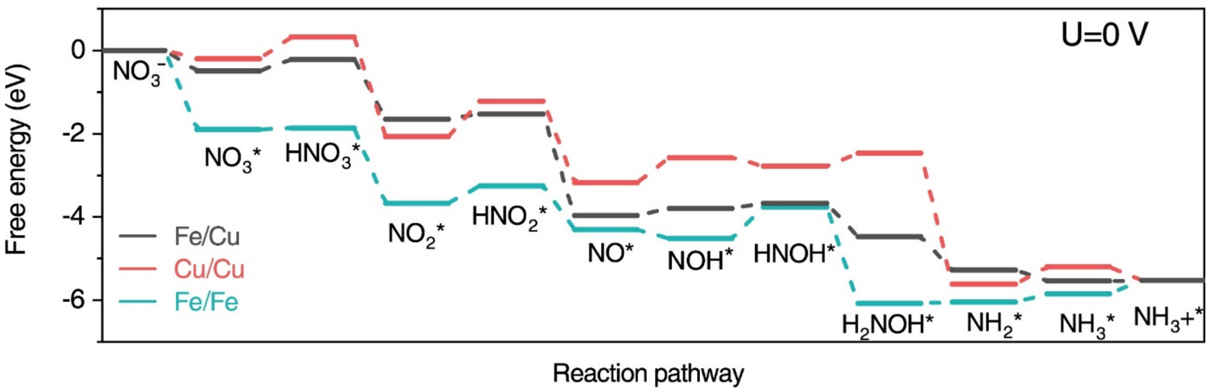

Supplementary Fig. 35 Free energy diagram of each intermediate state on the metal atom sites in Fe-Cu, Fe-Fe and Cu-Cu diatomic sites at U =0 V vs. RHE.

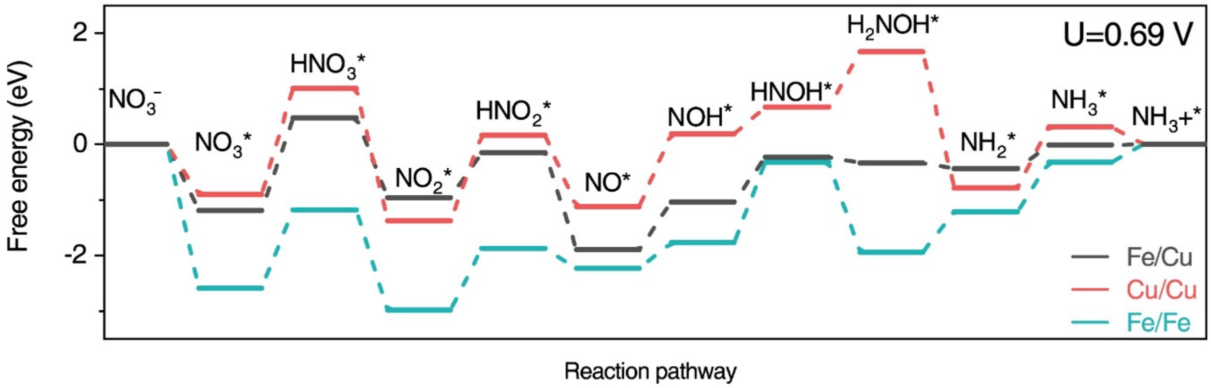

Supplementary Fig. 36 Free energy diagram of each intermediate state in Fe-Cu, Fe-Fe and Cu-Cu diatomic sites at U =0.69 V vs. RHE.

Supplementary Tables

Table 1. Elemental composition for the catalysts.

Sample	Iron (wt.%)	Copper (wt.%)
Fe/Cu-HNG	3.3	2.8
Fe-HNG	6.3	
Cu-HNG		5.9

Table 2. EXAFS structure parameters from the different samples.

Sample	K-edge	Shell	N	r/Å	$\sigma^2/10^{-3}Å^2$	R
Fe/Cu-HNG	Fe	Fe-Cu	0.87	2.26 ± 0.083	2.87 ± 0.35	0.003
		Fe-N	2.15	2.01 ± 0.041	4.32 ± 0.61	
	Cu	Fe-Cu	0.86	2.24 ± 0.036	3.14 ± 0.34	0.002
		Cu-N	2.33	2.05 ± 0.026	2.66 ± 0.43	
Fe-HNG	Fe	Fe-N	1.92	1.74 ± 0.028	3.85 ± 0.27	0.005
Cu-HNG	Cu	Cu-N	1.95	1.92 ± 0.013	6.50 ± 0.33	0.002
Fe/Fe-HNG	Fe	Fe-Fe	0.87	2.17 ± 0.017	5.24 ± 0.16	0.006
		Fe-N	2.13	1.89 ± 0.024	5.17 ± 0.30	
Cu/Cu-HNG	Cu	Cu-Cu	0.89	2.31 ± 0.034	3.90 ± 0.37	0.003
		Cu-N	2.10	1.95 ± 0.018	1.96 ± 0.15	
Fe-foil	Fe	Fe-Fe	8	2.45 ± 0.012	3.89 ± 0.16	0.003
Cu-foil	Cu	Cu-Cu	12	2.54 ± 0.022	8.30 ± 0.48	0.004

N: coordination number; *r*: bond length;

σ^2: Debye-Waller factor (disorder); *R*: R-factor.

Table 3. Reported catalysts for the nitrate reduction to ammonia.

Materials	Maximum FE to NH_3	Corresponding potential to NH_3	Maximum NH_3 yield rate[a]	Maximum energy consumption	Electrolyte conditions	Ref
Fe/Cu-NG	~92.51 %	−0.3 V vs RHE	1.08 mmol h^{-1} mg^{-1} −0.5 V vs RHE	8.76 Wh g_{NH3}^{-1} mg^{-1}	0.10 M KNO_3, 1.0 M KOH, pH=14	This work
Fe single atom	~75%	−0.66 V vs RHE	0.46 mmol h^{-1} cm^{-2} ~20 mg h^{-1} $mg_{cat.}^{-1}$ −0.85 V vs RHE	26.60 Wh g_{NH3}^{-1} cm^{-2}	0.50 M KNO_3, 0.10 M K_2SO_4, pH=7	2
Cu-PTCDA	85.9%	−0.4 V vs RHE	0.05 mmol h^{-1} cm^{-2} −0.6 V vs RHE	53.83 Wh g_{NH3}^{-1} cm^{-2}	36 mM NO_3^-, 0.1 mM PBS, pH=7	3
Single-site iron	~100%	After −0.3V vs RHE	0.16 mmol h^{-1} cm^{-2} −0.7 V vs RHE	28.37 Wh g_{NH3}^{-1} cm^{-2}	0.1 M KNO_3, 0.1 M KOH, pH=13	11
defective CuO	89%	−0.5 V vs RHE	0.33 mmol h^{-1} cm^{-2} −0.7 V vs RHE	68.00 Wh g_{NH3}^{-1} cm^{-2}	0.05 M KNO_3, 0.05 M H_2SO_4, pH=1	12
$Cu_{49}Fe_1$	94.5%	−0.74 V vs RHE	0.23 mmol h^{-1} cm^{-2} −0.74 V vs. RHE	28.01 Wh g_{NH3}^{-1} cm^{-2}	200 ppm KNO_3, 0.1 M K_2SO_4, pH=7	13
Fe-cyano NSs	90%	−0.5 V vs RHE	42.1 mg h^{-1} mg_{cat}^{-1} −0.5V vs RHE	12.32 Wh g_{NH3}^{-1} mg_{cat}^{-1}	0.10 M KNO_3, 1.0 M KOH, pH=14	14
Cu/Cu_2O	95.8%	−0.85 V vs RHE	0.245 mmol h^{-1} cm^{-2} −0.85 V vs RHE	64.92 Wh g_{NH3}^{-1} cm^{-2}	14.3 mM NO_3^-, 0.5 M $NaSO_4$, pH=7	15
TiO_{2-X}	85%	−1.6 V vs SCE	0.765 mg h^{-1} mg_{cat}^{-1} −1.6 V vs SCE	31.41 Wh g_{NH3}^{-1} mg_{cat}^{-1}	3.6 mM NO_3^-, 0.5 M $NaSO_4$, pH=7	16
Co/CoO NSAs	93.8%	−1.3 V vs SCE	0.19 mmol h^{-1} cm^{-2} −1.3 V vs SCE	31.94 Wh g_{NH3}^{-1} cm^{-2}	14.3 mM NO_3^-, 0.1 M $NaSO_4$, pH=7	17

Rh@Cu	93%	−0.2 V vs RHE	1.27 mmol h^{-1} cm^{-2} −0.4 V vs RHE	19.40 Wh g_{NH3}^{-1} cm^{-2}	0.1 M KNO_3, 0.1 M Na_2SO_4, pH=11.5	18
CoP NAs/CFC	~100%	−0.3 V vs RHE	9.56 mol h^{-1} m^{-2} −0.3 V vs RHE	19.79 Wh g_{NH3}^{-1} cm^{-2}	1.0 M $NaNO_3$, 1.0 M NaOH, pH=14	19
a-RuO_2	97.46 %	−0.35 V vs RHE	0.1158 mmol h^{-1} cm^{-2} −0.35 V vs RHE	32.10 Wh g_{NH3}^{-1} cm^{-2}	200 ppm $NaNO_3$, 0.5 M Na_2SO_4, pH=7	20
Bi-X_{red}	90.6%	−0.5 V vs RHE	46.5 g h^{-1} g_{cat}^{-1} −0.8 V vs RHE	13.09Wh g_{NH3}^{-1} mg_{cat}^{-1}	0.5 M KNO_3, 1.0 M KOH, pH=14	21
Cu single-atom catalyst	~88%	−1.2 V vs RHE	27.84 mg h^{-1} cm^{-2} −2.0 V vs RHE	42.47 Wh g_{NH3}^{-1} cm^{-2}	1000 ppm KNO_3, 0.5 M Na_2SO_4, pH=7	22
Cu-N-C SAC	84.7%	−1.0 V vs RHE	4.5 mg h^{-1} cm^{-2} −1.0 V vs RHE	23.28 Wh g_{NH3}^{-1} cm^{-2}	0.1 M KNO_3, 0.1 M KOH, pH=13	23
Ru-CuNW	96%	0.04 V vs RHE	76.5 mg h^{-1} cm^{-2} −0.135 V vs RHE	17.19 Wh g_{NH3}^{-1} cm^{-2}	2,000 ppm NO_3^-, 1 M KOH, pH=14	24
Co-SACs	92.0%	−0.69 V vs RHE	0.433 mg h^{-1} cm^{-2} −0.89 vs RHE	24.53 Wh g_{NH3}^{-1} cm^{-2}	100 mg/L NO_3^-, 0.02 M Na_2SO_4, pH=7	25
Copper/cobalt-based catalysts	93.3%	−0.175 V vs RHE	1.17 mmol h^{-1} cm^{-2} −0.175 V vs RHE	26.82 Wh g_{NH3}^{-1} cm^{-2}	0.01 M KNO_3, 0.1 M KOH, pH=13	26
Meso-PdN Alloy	96.1%	−0.7 V vs RHE	4.6 mg h^{-1} mg_{cat}^{-1} −0.8 V vs RHE	11.3 Wh g_{NH3}^{-1} mg_{cat}^{-1}	5.0 mM KNO_3, 0.1 M Na_2SO_4, pH=7	27
Fe_2TiO_5 nanofiber	87.6%	−1.0 V vs RHE	0.73 mmol h^{-1} mg_{cat}^{-1} −1.0 V vs RHE	6.29 Wh g_{NH3}^{-1} mg_{cat}^{-1}	0.1 M $NaNO_3$, PBS solution, pH= 7.4	28

MP-Cu	99.8%	−0.3 V vs RHE	0.543 mmol h^{-1} cm^{-2} −0.3 V vs RHE	24.86 Wh g_{NH3}^{-1} cm^{-2}	0.05 M NO_3^-, 1 M KOH, pH=14	29
CoP-CNS	93.3%	−0.33 V vs RHE	3.09 mmol h^{-1} cm^{-2} −1.03 V vs. RHE	29.79 Wh g_{NH3}^{-1} cm^{-2}	1.0 M $NaNO_3$, 1 M NaOH, pH=14	30
$NiCo_2O_4$ Nanowire Array	99.0%	−0.3 V vs RHE	0.972 mmol h^{-1} cm^{-2} −0.6 V vs. RHE	25.03 Wh g_{NH3}^{-1} cm^{-2}	0.1 M $NaNO_3$, 0.1 M NaOH, pH=13	31
$Pt_{0.9}/Ce_{0.5}$-SS	94.12 %	−0.5 V vs RHE	0.59 mmol h^{-1} cm^{-2} −0.8 V vs. RHE	38.45 Wh g_{NH3}^{-1} cm^{-2}	0.1 M KNO_3, 0.5 M Na_2SO_4, pH=11.5 (adjusted)	32
SN Co-Li^+/PCNF	72.7%	−0.94 V vs RHE	0.71 mmol h^{-1} cm^{-2} −0.94 V vs. RHE	44.95 Wh g_{NH3}^{-1} cm^{-2}	0.5 M NO_3^-, 0.5 M Na_2SO_4, pH=7	33
Au1Cu	98.7%	−0.2 V vs RHE	0.555 mg h^{-1} cm^{-2} −0.2 V vs. RHE	64.41 Wh g_{NH3}^{-1} cm^{-2}	7.14 mM NO_3^-, 0.1 M KOH, pH=13	34
Co_3O_4 nanotubes	99.5%	−0.6 V vs RHE	35 mg h^{-1} cm^{-2} −1.2 V vs RHE	27.08 Wh g_{NH3}^{-1} cm^{-2}	0.1 M KNO_3, 0.5 M K_2SO_4, pH=7	35
single-atom Ru sites	72.8%	−0.6 V vs RHE	0.15 mmol h^{-1} cm^{-2} −0.6 V vs RHE	53.82 Wh g_{NH3}^{-1} cm^{-2}	0.5 M NO_3^-, 1.0 M KOH, pH=14	36
RuO_x/Pd	98.6%	−0.5 V vs RHE	23.5 mg h^{-1} cm^{-2} −0.5 V vs RHE	21.81 Wh g_{NH3}^{-1} cm^{-2}	0.1 M KNO_3, 1 M KOH, pH=14	37
$Ni_3Co_6S_8$	86.3%	−0.4 V vs RHE	2.4 mg h^{-1} cm^{-2} −0.4 V vs RHE	54.33 Wh g_{NH3}^{-1} cm^{-2}	50 mg/L NO_3^-, 1 M KOH, pH=14	38
Bi1Pd	99.6%	−0.6 V vs RHE	47 mg h^{-1} cm^{-2} −0.7 V vs RHE	27.72 Wh g_{NH3}^{-1} cm^{-2}	0.1 M NO_3^-, 1 M KOH, pH=14	39
Fe-V_2O_5	97.1%	−0.7 V vs RHE	16 mg h^{-1} cm^{-2} −0.8 V vs RHE	26.64 Wh g_{NH3}^{-1} cm^{-2}	0.1 M NO_3^-, 1 M KOH, pH=14	40

amorphous graphene	95%	−0.93 V vs RHE	3 mg h^{-1} cm^{-2} −0.93 V vs RHE	21.60 Wh g_{NH3}^{-1} mg_{cat}^{-1}	1 M $NaNO_3$, pH= 7	41
MBene	96.8%	−0.6 V vs RHE	35 mg h^{-1} cm^{-2} −0.8 V vs RHE	29.58 Wh g_{NH3}^{-1} mg_{cat}^{-1}	0.1 M NO_3^-, 1 M KOH, pH=14	42
NiO_4-CCP	94.7%	−0.7 V vs RHE	1.83 mmol h^{-1} cm^{-2} −0.9 V vs RHE	7.75 Wh g_{NH3}^{-1} mg_{cat}^{-1}	0.5 M NO_3^-, 1 M Na_2SO_4, pH=7	43
Ru_xCu_y/rGO	98%	−0.05 V vs RHE	0.38 mmol h^{-1} cm^{-2} −0.05 V vs RHE	20.80 Wh g_{NH3}^{-1} mg_{cat}^{-1}	0.1 M NO_3^-, 1 M KOH, pH=14	44
ZnSA-MNC	94.8 %	−1.0 V vs RHE	0.32 mmol h^{-1} cm^{-2} −1.0 V vs RHE	11.27 Wh g_{NH3}^{-1} mg_{cat}^{-1}	0.5 mM $NaNO_3$, 0.1 M Na_2SO_4, pH=7	45
Pd/NF	80%	−1.0 V vs RHE	1.52 mmol h^{-1} cm^{-2} −1.4 V vs RHE	24.43 Wh g_{NH3}^{-1} mg_{cat}^{-1}	0.1 M $NaNO_3$, 0.5 M Na_2SO_4, pH=7	46
Cu-doped Fe_3O_4	100%	−0.6 V vs RHE	7.18 mg h^{-1} cm^{-2} −0.6 V vs RHE	14.01 Wh g_{NH3}^{-1} mg_{cat}^{-1}	0.1 M KNO_3, 0.1 M KOH, pH=13	47
NiPr-TPA-COF	90%	−1.38 V vs SCE	2.5 mg h^{-1} cm^{-2} −1.46 V vs SCE	24.92 Wh g_{NH3}^{-1} mg_{cat}^{-1}	0.1 M KNO_3, 0.5 M K_2SO_4, pH=7	48
Rh NFs	95%	0.2 V vs RHE	0.253 mg h^{-1} cm^{-2} 0.2 V vs RHE	10.18 Wh g_{NH3}^{-1} mg_{cat}^{-1}	0.1 M NO_3^-, 0.1 M Na_2SO_4 pH=11.5 (adjusted)	49
Ni_3Fe–CO_3 LDH	96.8%	−0.2 V vs RHE	1.261 mg h^{-1} cm^{-2} −0.2 V vs RHE	28.35 Wh g_{NH3}^{-1} mg_{cat}^{-1}	5 mM KNO_3, 1 M KOH, pH=14	50

a: Maximum yield rate at corresponding voltage recorded in the literature;

Supplementary References

1. Gong, Z. *et al.* Regulating surface oxygen species on copper (I) oxides via plasma treatment for effective reduction of nitrate to ammonia. *Appl. Catal. B: Environ.* **305**, 121021 (2022).
2. Wu, Z.-Y. *et al.* Electrochemical ammonia synthesis via nitrate reduction on Fe single atom catalyst. *Nat. Commun.* **12**, 2870 (2021).
3. Chen, G.-F. *et al.* Electrochemical reduction of nitrate to ammonia via direct eight-electron transfer using a copper–molecular solid catalyst. *Nat. Energy* **5**, 605-613 (2020).
4. Kresse, G. & Furthmüller, J. Efficient iterative schemes for ab initio total-energy calculations using a plane-wave basis set. *Phys. Rev. B* **54**, 11169-11186 (1996).
5. Kresse, G. & Joubert, D. From ultrasoft pseudopotentials to the projector augmented-wave method. *Phys. Rev. B* **59**, 1758-1775 (1998).
6. Hammer, B., Hansen, L. B. & Nørskov, J. K. Improved adsorption energetics within density-functional theory using revised Perdew-Burke-Ernzerhof functionals. *Phys. Rev. B* **59**, 7412-7421 (1999).
7. Wang, V. *et al.* VASPKIT: A user-friendly interface facilitating high-throughput computing and analysis using VASP code. *Comput. Phys. Commun.* **267**, 108033 (2021).
8. Mostofi, A. A. *et al.* wannier90: A tool for obtaining maximally-localised Wannier functions. *Comput. Phys. Commun.* **178**, 685-699 (2008).

9 Zhou, Q. *et al.* Nitrogen-doped CoP electrocatalysts for coupled hydrogen evolution and sulfur generation with low energy consumption. *Adv. Mater.* **30**, 1800140 (2018).

10. Calle-Vallejo, F., Huang, M., Henry, J. B., Koper, M. T. & Bandarenka, A. S. Theoretical design and experimental implementation of Ag/Au electrodes for the electrochemical reduction of nitrate. *Phys. Chem. Chem. Phys.* **15**, 3196-3202 (2013).

11. Li, P., Jin, Z., Fang, Z. & Yu, G. A single-site iron catalyst with preoccupied active centers that achieves selective ammonia electrosynthesis from nitrate. *Energy Environ. Sci.* **14**, 3522-3531 (2021).

12. Daiyan, R. *et al.* Nitrate reduction to ammonium: from CuO defect engineering to waste NO_x-to-NH_3 economic feasibility. *Energy Environ. Sci.* **14**, 3588-3598 (2021).

13. Wang, C. *et al.* Metasequoia-like nanocrystal of iron-doped copper for efficient electrocatalytic nitrate reduction into ammonia in neutral media. *ChemSusChem* **14**, 1825-1829 (2021).

14. Fang, Z. *et al.* Porous two-dimensional iron-cyano nanosheets for high-rate electrochemical nitrate reduction. *ACS Nano* **16**, 1, 1072-1081 (2022).

15. Wang, Y. *et al.* Unveiling the activity origin of a copper-based electrocatalyst for selective nitrate reduction to ammonia. *Angew. Chem. Int. Ed.* **59**, 5350-5354 (2020).

16. Jia, R. *et al.* Boosting selective nitrate electroreduction to ammonium by constructing oxygen vacancies in TiO_2. *ACS Catal.* **10**, 3533-3540 (2020).

17. Yu, Y. *et al.* Promoting selective electroreduction of nitrates to ammonia over electron-deficient Co modulated by rectifying Schottky contacts. *Sci. China Chem.* **63**, 1469-1476 (2020).

18. Liu, H. *et al.* Efficient electrochemical nitrate reduction to ammonia with copper-supported rhodium cluster and single-atom catalysts. *Angew. Chem. Int. Ed. Engl.* **61**, 202202556 (2022).

19. Ye, S. *et al.* Elucidating the activity, mechanism and application of selective electrosynthesis of ammonia from nitrate on cobalt phosphide. *Energy Environ. Sci.* **15**, 760-770 (2022).

20. Wang, Y. *et al.* Structurally disordered RuO_2 nanosheets with rich oxygen vacancies for enhanced nitrate electroreduction to ammonia. *Angew. Chem. Int. Ed. Engl.* **61**, 202202604 (2022).

21. Zhang, N. *et al.* Governing interlayer strain in bismuth nanocrystals for efficient ammonia electrosynthesis from nitrate reduction. *ACS Nano* **16**, 4795-4804 (2022).

22. Cheng, X. F. *et al.* Coordination symmetry breaking of single-atom catalysts for robust and efficient nitrate electroreduction to ammonia. *Adv. Mater.* **34**, 2205767 (2022).

23. Yang, J. *et al.* Potential-driven restructuring of cu single atoms to nanoparticles for boosting the electrochemical reduction of nitrate to ammonia. *J. Am. Chem. Soc.* **144**, 12062-12071 (2022).

24. Chen, F. Y. et al. Efficient conversion of low-concentration nitrate sources into ammonia on a Ru-dispersed Cu nanowire electrocatalyst. *Nat. Nanotechnol.* **17**, 759-767 (2022).

25. Li, J. *et al.* Boosted ammonium production by single cobalt atom catalysts with high faradic efficiencies. *Proc. Natl. Acad. Sci. USA* **119**, 2123450119 (2022).

26. He, W. *et al.* Splicing the active phases of copper/cobalt-based catalysts achieves high-rate tandem electroreduction of nitrate to ammonia. *Nat. Commun.* **13**, 1129 (2022).

27. Sun, L. & Liu, B. Mesoporous PdN alloy nanocubes for efficient electrochemical nitrate reduction to ammonia. *Adv. Mater.* **35**, 2207305 (2023).

28. Du, H. *et al.* Durable electrocatalytic reduction of nitrate to ammonia over defective pseudobrookite Fe_2TiO_5 nanofibers with abundant oxygen vacancies. *Angew. Chem. Int. Ed. Engl.* **62**, 202215782 (2023).

29. Wen, W. *et al.* Metastable phase Cu with optimized local electronic state for efficient electrocatalytic production of ammonia from nitrate. *Adv. Funct. Mater.* **33**, 2212236 (2022).

30. Fan, K. *et al.* Active hydrogen boosts electrochemical nitrate reduction to ammonia. *Nat. Commun.* **13**, 7958 (2022).

31. Liu, Q. *et al.* Ambient ammonia synthesis via electrochemical reduction of nitrate enabled by $NiCo_2O_4$ nanowire array. *Small* **18**, 2106961 (2022).

32. Chen, D. *et al.* Tailored p-orbital delocalization by diatomic Pt-Ce induced interlayer spacing engineering for highly-efficient ammonia electrosynthesis. *Adv. Energy Mater.* **13**, 2203201 (2022).

33. Mi, L. *et al.* Achieving synchronization of electrochemical production of ammonia from nitrate and ammonia capture by constructing a "two-in-one" flow cell electrolyzer. *Adv. Energy Mater.* **12**, 202202247 (2022).

34. Zhang, Y. *et al.* Electrocatalytic nitrate reduction to ammonia on defective Au1Cu (111) single-atom alloys. *Appl. Catal. B: Environ.* **310**, 121346 (2022).

35. Liu, D. *et al.* Electrocatalytic reduction of nitrate to ammonia on low-cost manganese-incorporated Co_3O_4 nanotubes. *Appl. Catal. B: Environ.* **324**, 122293 (2023).

36. Ke, Z. *et al.* Selective NO_x^- electroreduction to ammonia on isolated Ru sites. *ACS Nano* **17**, 3483-3491 (2023).

37. Li, X. *et al.* Sub-nm RuOx clusters on Pd metallene for synergistically enhanced nitrate electroreduction to ammonia. *ACS Nano* **17**, 1081-1090 (2023).

38. Tao, W. *et al.* Engineering sulfur vacancies optimization in $Ni_3Co_6S_8$ nanospheres toward extraordinarily efficient nitrate electroreduction to ammonia. *Appl. Catal. B: Environ.* **324**, 122193 (2023).

39. Chen, K. *et al.* Single-atom Bi alloyed pd metallene for nitrate electroreduction to ammonia. *Adv. Funct. Mater.* 2209890 (2023).

40. Zhang, N. *et al.* Lewis acid Fe-V pairs promote nitrate electroreduction to ammonia. *Adv. Funct. Mater.* 2211537 (2023).

41. Huang, L. *et al.* Direct synthessis of ammonia from nitrate on amorphous graphene with near 100% efficiency. *Adv. Mater*. 2211856 (2023).

42. Zhang, G. *et al.* Tandem electrocatalytic nitrate reduction to ammonia on mbenes. *Angew. Chem. Int. Ed. Engl*. 202300054 (2023).

43. Zhang, Y. *et al.* Conjugated coordination polymer as a new platform for efficient and selective electroreduction of nitrate into ammonia. *Adv. Mater*. 2209855 (2023).

44. Gao, W. *et al.* Alloying of Cu with Ru enabling the relay catalysis for reduction of nitrate to ammonia. *Adv. Mater*. 2202952 (2023).

45. Zhao, J. *et al.* Zn single atom on N-doped carbon: Highly active and selective catalyst for electrochemical reduction of nitrate to ammonia. *Chem. Eng. J.* **452**, 139533 (2023).

46. Guo, H. *et al.* Self-supported Pd nanorod arrays for high-efficient nitrate electroreduction to ammonia. *Small* 2207743 (2023).

47. Wang, J. *et al.* Cu-doped iron oxide for the efficient electrocatalytic nitrate reduction reaction. *Nano Lett.* **23**, 1897-1903 (2023).

48. Lv, F. *et al.* Near-unity electrochemical conversion of nitrate to ammonia on crystalline nickel porphyrin-based covalent organic frameworks. *Energ. Environ. Sci.* **16**, 201-209 (2023).

49. Liu, H. *et al.* Low-coordination rhodium catalysts for an efficient electrochemical nitrate reduction to ammonia. *ACS Catal.* **13**, 1513-1521 (2023).

50. Kim, K.-H. *et al.* Energy-efficient electrochemical ammonia production from dilute nitrate solution. *Energ. Environ. Sci.* **16**, 663-672 (2023).
